# Guanylate-binding proteins induce apoptosis of leukemia cells by regulating MCL-1 and BAK

**DOI:** 10.1038/s41389-021-00341-y

**Published:** 2021-07-22

**Authors:** Yongyang Luo, Hanyong Jin, Je Hyeong Kim, Jeehyeon Bae

**Affiliations:** 1 School of Pharmacy, Chung-Ang University, Seoul, 06974 Korea; 2 Department of Life Science, Chung-Ang University, Seoul, 06974 Korea; 3 Division of Pulmonology, Department of Internal Medicine, Korea University Ansan Hospital, Ansan, 15355 Korea

**Keywords:** Haematological cancer, Cell death

## Abstract

Interferon-inducible guanylate-binding proteins (GBPs) are well-known for mediating host-defense mechanisms against cellular pathogens. Emerging evidence suggests that GBPs are also implicated in tumorigenesis; however, their underlying molecular mechanism is still unknown. In this study, we identified that GBP1 and GBP2 interact with MCL-1, the key prosurvival member of the BCL-2 family, via its BH3 domain. GBPs induce caspase-dependent apoptosis in chronic myeloid leukemia (CML) and acute myeloid leukemia (AML) cells, where the proapoptotic BCL-2 member, BAK, is an indispensable mediator. In particular, GBP2 completely inhibited the MCL-1-mediated promotion of the survival of CML cells through competitive inhibition, resulting in BAK liberation from MCL-1. Concurrently, GBP2 dramatically upregulates BAK expression via its inhibition of the PI3K/AKT pathway. Moreover, paclitaxel upregulates GBP2 expression, and paclitaxel-induced apoptotic activity was distinctively compromised by knockout of *GBP2* in CML cells. Bioinformatics analyses of leukemia databases revealed that transcripts of *GBPs* were generally downregulated in leukemia patients and that GBPs were favorable prognosis markers. Thus, these findings provide molecular evidence of GBPs as apoptosis-inducing proteins of leukemia cells and suggest that GBPs are attractive targets for the development of chemotherapeutics.

## Introduction

Leukemia is characterized by the uncontrolled proliferation of abnormal white blood cells and is divided into four main subtypes: acute myeloid leukemia (AML), chronic myeloid leukemia (CML), acute lymphoblastic leukemia (ALL), and chronic lymphocytic leukemia (CLL) [1]. Among these, AML has the highest incidence and mortality rate, and CML accounts for 15% of newly diagnosed leukemia cases in Western society [2]. The major genetic hallmark of CML is the reciprocal chromosomal translocation between chromosomes 9 and 22, which is the Philadelphia chromosome, generating the BCR-ABL1 oncoprotein that exhibits the abnormal tyrosine kinase properties of myeloid cells [3]. Although the CML survival rate has increased by the advent of tyrosine kinase inhibitors (TKIs), such as imatinib, the development of drug resistance in CML patients treated with TKIs in addition to the long-term tolerability and side effects of the TKIs treatment limit its therapeutic usability [4]. While the etiology of AML is more complicated because of the involvement of a broad range of genetic alterations, cytotoxic induction chemotherapy is the first-line treatment option at present [5].

Dysregulated apoptosis is often associated with cancerous tumors. The BCL-2 protein family is composed of central regulators of apoptosis and consists of both antiapoptotic (BCL-2, MCL-1, BCL-xL, BCL-w, and BCL2A1) and proapoptotic members to coordinate the survival and death of cells by dimer formation between specific BCL-2 members [6]. Upon death signaling, BAK, BAX, and/or BOK (death effector molecules of proapoptotic BCL-2 family members) form oligomers leading to mitochondrial outer membrane permeabilization (MOMP), which is followed by the release of apoptotic molecules, including cytochrome *c*, which subsequently activates caspases [7]. *Myeloid cell leukemia 1* (*MCL-1*) has been identified as an early-induced gene during the differentiation of human myeloblastic leukemia cells [8], and its protein is a distinct antiapoptotic member of the BCL-2 family crucial for the survival of diverse types of cells [9]. MCL-1 promotes cell survival via its ability to bind and sequester proapoptotic BCL-2 proteins, including BAK, BAX, and BIM [10]. MCL-1 is known as a BCR/ABL-dependent survival factor in CML and a key factor that contributes to CML progression and therapeutic outcome [11]. In addition, the essential and crucial role of MCL-1 for the development and growth of AML has been well demonstrated [12]. Therefore, understanding the molecular regulatory network of MCL-1 is of tremendous importance to be able to control the survival-promoting MCL-1 function in myeloid leukemia.

GBP2, a member of the highly conserved guanylate-binding protein (GBP) family, belongs to the dynamin superfamily of large GTPases [13]. In humans, seven GBPs (GBP1–7) have been found positioned in a single cluster on chromosome 1 [14]. GBP2, similar to GBP1, consists of an N-terminal globular domain harboring the GTPase activity and a C-terminal helical domain composed of seven alpha-helices [15]. GBP proteins are induced by inflammatory cytokines, including IFN α/β/γ, tumor necrosis factor-α, and toll-like receptor agonists [16]. The biological roles of the GBP protein family in living organisms have largely remained unknown except for their function in mediating host-defense mechanisms against cellular pathogens [17]. Recently, emerging clinical evidence implies that GBP proteins may also play an important role during tumor development [18–22], but the molecular mechanism that links GBPs and cancer is still unknown.

Here, we discovered GBP1 and GBP2 as interacting proteins of MCL-1 and demonstrated a novel function of GBPs as mitochondrial apoptosis-inducing molecules in AML and CML cells. Furthermore, the underlying molecular mechanism of GBP1 and GBP2 as apoptosis inducers, their clinical implications in leukemia, and their efficacy as potential chemotherapeutic agents were investigated.

## Results

### GBP1 and GBP2 are MCL-1-binding proteins in leukemic cells

**Fig. 1:**
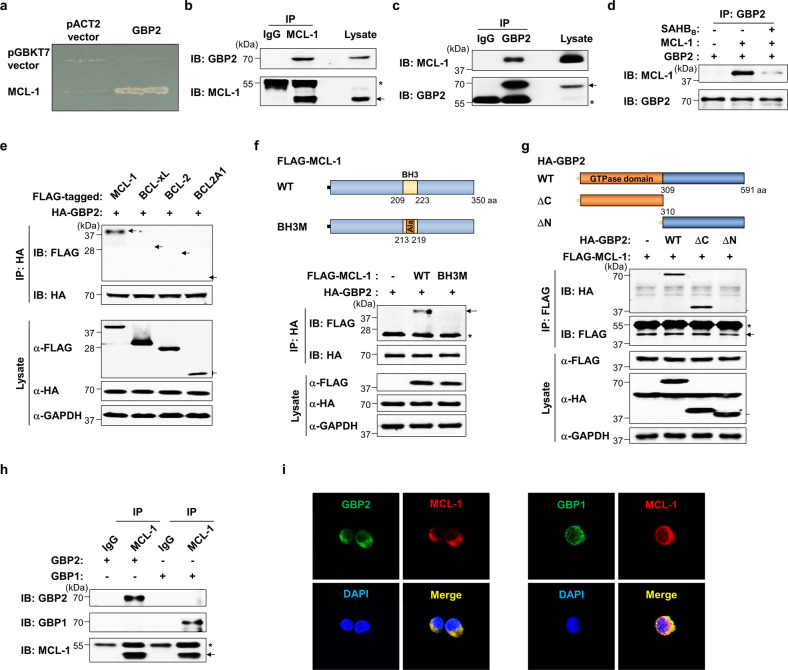
GBP1 and GBP2 specifically interact with MCL-1. **a** Yeast growth was demonstrated in colonies expressing both *GBP2* and *MCL-1* fused to the GAL4 DNA activation domain and binding domain, respectively. **b**, **c** The endogenous protein–protein interaction between GBP2 and MCL-1 was confirmed using immunoprecipitations followed by western blot analyses using indicated antibodies in K562 cells (**b**) and HL-60 cells (**c**). IgG was used as an immunoprecipitation control. **d** In vitro binding of GBP2 and MCL-1 was determined using purified recombinant proteins (0.5 µg) of GBP2 and MCL-1 with or without the presence SAHB_B_ (10 µM), a BH3-specific inhibitor of MCL-1. Subsequently, immunoprecipitation and immunoblotting were performed with indicated antibodies. **e** The interaction of GBP2 with prosurvival members of the BCL-2 family was examined by immunoprecipitation following cotransfection of 3 µg of plasmids encoding HA-GBP2 and FLAG-BCL-2 family members in K562 cells. Arrows indicate the expected positions of respective proteins. GAPDH was included as a loading control. **f**, **g** Schematic representations of the plasmids encoding WT and mutants of MCL-1 (**f**) and GBP2 (**g**), which were generated to determine the binding domains. 293 T cells (2 × 10^6^) were cotransfected with indicated plasmids. After transfection for 24 h, cell lysates were immunoprecipitated with anti-HA (**f**) or anti-FLAG antibodies (**g**) followed by immunoblotting with the indicated antibodies. **h** The interaction between GBP1 and MCL-1 was assessed in K562 cells after the transfection of plasmids (3 µg) encoding GBP1 or GBP2 followed by immunoprecipitation. **i** The intracellular localization and colocalization of endogenous MCL-1 with GBP1 or GBP2 in K562 cells were visualized by fluorescent confocal microscopy. For all immunoblot images presented throughout this manuscript, the membrane was cut into pieces according to the estimated molecular weight of proteins of interest and probed with the indicated antibodies. All cropped blots were run under the same experimental conditions.

To discover MCL-1-interacting proteins, we used the full-length human MCL-1 as bait and performed yeast two-hybrid screening using the human ovary cDNA library as described in our previous study [23]. Human GBP2 was identified, and the specific interaction between GBP2 and MCL-1 was confirmed in yeast (Fig. 1a). We further verified the interaction of endogenous GBP2 and MCL-1 proteins in both K562 (a CML cell line) and HL-60 (an AML cell line) by immunoprecipitation followed by western blot analysis (Fig. 1b, c). In addition, the direct association of GBP2 and MCL-1 was demonstrated by in vitro co-immunoprecipitation of recombinant GBP2 and MCL-1 proteins (Fig. 1d and Supplementary Fig. 1a) and further validated using SAHB_B_, a specific MCL-1 inhibitor [24], as their interaction affinity was significantly diminished by the presence of SAHB_B_ (Fig. 1d). GBP2 specifically and selectively interacted with antiapoptotic BCL-2 family protein MCL-1, but not with other prosurvival BCL-2 family proteins, such as BCL-xL, BCL-2, and BCL2A1 (Fig. 1e).

To define the binding regions of MCL-1 and GBP2, we generated a BH3 mutant (BH3M), in which seven conserved amino acids of the BH3 domain were substituted to alanine (Fig. 1f), and two truncated mutants of GBP2 (GBP2-ΔC and ΔN; Fig. 1g). GBP2 failed to interact with the MCL-1 ∆BH3, suggesting that the BH3 domain is required for GBP2 interaction with MCL-1 (Fig. 1f). The GBP2 ΔN mutant lacking the N-terminus GTPase domain did not interact with MCL-1, whereas the 309 amino acid fragment at the N-terminal (GBP2 ΔC) was sufficient for the interaction (Fig. 1g). Moreover, we observed that GBP1, a member of the GBP family that has 83.4% sequence identity to GBP2, interacted with MCL-1 in K562 (Fig. 1h) and HL-60 cells (Supplementary Fig. 1b). Fluorescent confocal microscopic images showed that both GBP1 and GBP2 proteins are colocalized with MCL-1 in the cytoplasm of K562 cells (Fig. 1i). We also performed immunoprecipitation experiments using mutants of MCL-1 and GBP1. Similar to what we observed with GBP2, the BH3 domain of MCL-1 and the GTPase domain of GBP1 are required for the interaction of MCL-1 with GBP1 (Supplementary Fig. 2a, b).

### GBP1 and GBP2 induce caspase-dependent apoptosis of leukemia cells

**Fig. 2:**
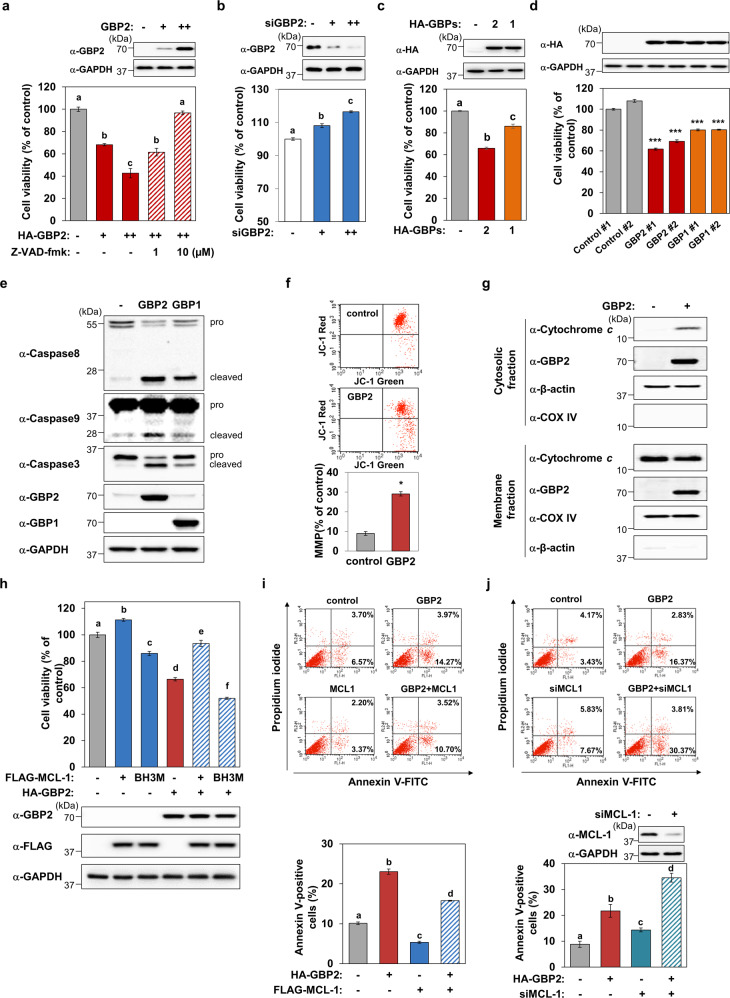
GBP1 and GPB2 induce apoptotic cell death. **a** Cell viability assay was performed in HA-GBP2-transfected cells after treating with a solvent vehicle (0.02% DMSO) or Z-VAD-FMK (1 or 10 μΜ). The expression levels of GBP2 were assessed by western blotting. **b** The effect of *GBP2* silencing on the cell viability of K562 cells was determined following the transfection of siRNAs for *GBP2* (100 or 200 nM). Efficient knockdown of *GBP2* is demonstrated by immunoblot analysis. Different letters denote statistically significant differences (*P* < 0.01). **c** K562 cells were transfected with HA-tagged GBP2 or GBP1 expression plasmids (GBP2: 3 µg, GBP1: 1.8 µg) for 24 h following with cell viability assay (bottom). Different letters denote statistically significant differences (*P* < 0.001). The expression levels of GBP1/2 were assessed by western blotting. **d** The differences in cellular viability were examined in HA-GBP2- or HA-GBP1-stably overexpressing K562 cells (1 × 10^4^) after 24 h of culture. Asterisks indicate statistically significant differences compared to WT cells (***P < 0.001). Data are shown as mean ± SEM of three independent experiments. **e** GBP1- and GBP2-induced activations of Caspase 3, 8, and 9 were determined by western blot analyses in GBPs-overexpressed K562 cells. **f** GBP2-induced mitochondrial membrane permeability (MMP) was assessed by FACS analysis after the incubation of transfected K562 cells with JC-1. Representative (upper panel) and quantified (lower panel) MMP transition in empty or GBP2-transfected cells are shown. Asterisks indicate statistically significant differences (*P* < 0.05). Data are presented as the mean ± SEM of three independent experiments. **g** Cytochrome *c* levels in the cytosolic or mitochondrial fractions were analyzed in GBP2-transfected K562 cells by western blot analyses. The fractionation quality was demonstrated using anti-COX IV and anti-β-actin antibodies. **h** K562 cells (1 × 10^4^) were cotransfected with GBP2 expression plasmid (30 ng) and indicated MCL-1 plasmids (30 ng) for 24 h, and then cell viability was measured. Different letters denote statistically significant differences (*P* < 0.05). Data are shown as the mean ± SEM of three independent experiments performed in triplicate. The expression levels of GBP2, MCL-1 were determined by western blot using indicated antibodies. Representative blots are shown. **i**, **j** Cell apoptosis was analyzed by flow cytometry in K562 cells cotransfected with indicated plasmids (3 µg, **i**) or siRNAs (200 nM, **j**). Representative (upper panel) and quantitative (lower panel) data are shown. The percentages of early apoptosis (Annexin V-positive, PI-negative) cells and late apoptosis cells (both Annexin V- and PI-positive) are indicated. Efficient MCL-1 knockdown was determined by immunoblot analysis. The histogram data are presented as the mean ± SEM of three independent experiments performed in duplicate. Different letters denote statistically significant differences (*P* < 0.05).

To investigate the functions of GBP proteins in leukemic cells, we performed cell viability assays after modulating their expression levels. The overexpression of GBP1 or GBP2 induced cell death, which was completely inhibited by Z-VAD-FMK, the pan-caspase inhibitor (Fig. 2a and Supplementary Fig. 3a). Silencing of GBP2 promoted the viability of K562 cells (Fig. 2b). When similar amounts of GBP1/2 proteins were overexpressed, both GBP1 and GBP2 induced cell death (Fig. 2c). We generated two independent K562 cell lines that stably overexpress either GBP1 or GBP2 and performed functional analyses using these cells. As presented in Fig. 2d, both the GBP1- or GBP2-stably expressing cell lines showed significantly lower levels of cell viability (Fig. 2d) than the control cell lines, which is consistent with the effects observed upon the ectopic expression of these GBPs. Cell death activities of GBP1 and GBP2 were further confirmed in GBPs stably expressed K562 cells (Fig. 2d). Similar extents of HL-60 cell death activity induced by GBP1 and GBP2 were observed (Supplementary Fig. 3b). Both GBP proteins activated the initiator caspases 8 and 9, and the effector caspase 3 as shown by western blot analysis (Fig. 2e). GBP2 significantly induced MMP (Fig. 2f) and the release of mitochondrial cytochrome *c* into the cytosol (Fig. 2g). In addition, the percentages of apoptotic cells in the sub-G1 fraction were also analyzed, and the results indicated that both GBP1 and GBP2 induced apoptotic cell death (Supplementary Fig. 3c). In contrast, the overexpression of GBP1 or GBP2 did not significantly affect the cell cycle progression of K562 cells (Supplementary Fig. 3d). Collectively, these results demonstrate that GBP1 and GBP2 induce the mitochondrial apoptotic death of leukemia cells.

### GBP2 inhibits the survival of leukemic cells induced by MCL-1

We further assessed the function of GBP proteins and MCL-1 in regulating leukemic cell survival and death. The overexpression of MCL-1 promoted cell survival, while its BH3 mutant induced the death of K562 cells (Fig. 2h). MCL-1 failed to promote cell survival when either GBP1 or GBP2 were overexpressed (Fig. 2h and Supplementary Fig. 3e). Concurrently, the induced cell death activities of GBP1 or GBP2 were significantly attenuated by MCL-1 overexpression, whereas the BH3-mutated MCL-1 that lacked GBP2-binding capacity (Fig. 1f) failed to inhibit the activity of GBP2 (Fig. 2h and Supplementary Fig. 3e). The number of annexin V-positive apoptotic cells was clearly increased by GBP2 expression, and the antiapoptotic action of MCL-1 was completely prevented by GBP2 in K562 cells (Fig. 2i). In addition, MCL-1 depletion increased the number of apoptotic cells, and this number was further augmented by GBP2 overexpression (Fig. 2j).

### BAK is an essential mediator of GBP2-induced cell death

**Fig. 3:**
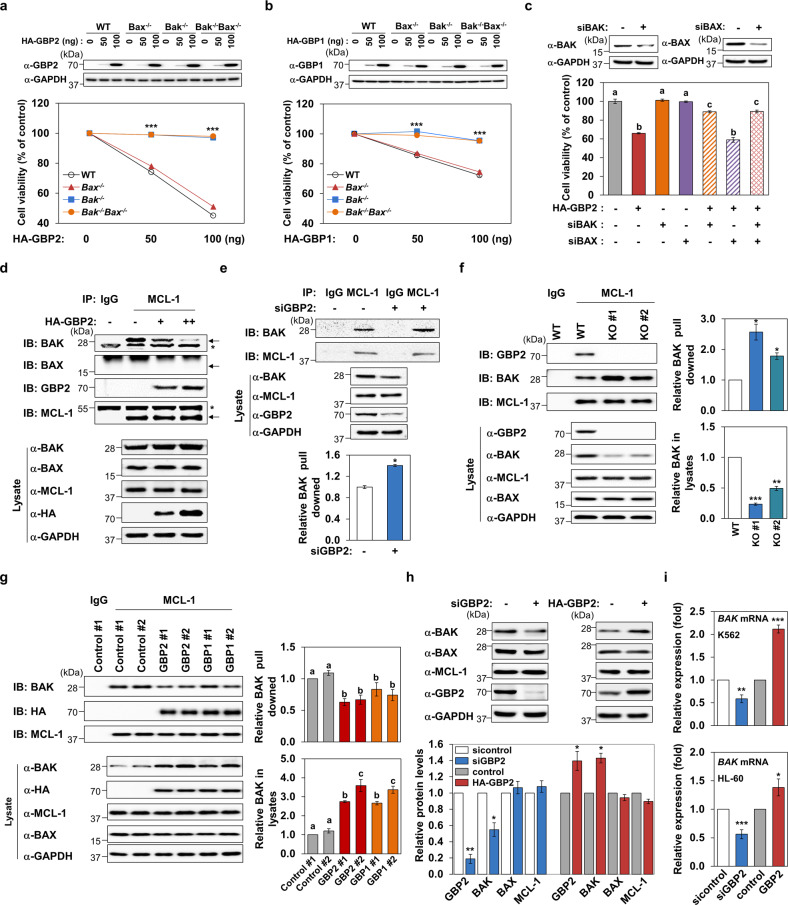
BAK is an essential mediator of GBP1- and GBP2-induced cell death, and GBP2 competitively inhibits MCL-1-BAK interaction. **a**, **b** The effects of GBPs on cell viability were determined in MEF cells of WT, *Bak*^*−/*−^, *Bax*^*−/−*^, and *Bax*^−*/−*^*Bak*^*−/−*^. Asterisks indicate statistically significant values compared with those of WT cells (***P < 0.001). Data are presented as the mean ± SEM of three independent experiments performed in triplicate. Cell lysates were subjected to western blot to confirm similar ectopic expression of GBP2 and GBP1 in all cell lines. **c** The effects of *BAK* or *BAX* knockdown on GBP2-induced cell death activity was determined in K562 cells. Efficient silencing of *BAK* and *BAX* was confirmed by western blot analyses. Different letters denote statistically significant differences (*P* < 0.01). Data are presented as the mean ± SEM of three independent experiments performed in triplicate. **d**, **e** Effects of GBP2 overexpression (**d**) or silencing (**e**) on the complex formation between MCL-1 and BAK were evaluated by immunoprecipitation following transfection of K562 cells with HA-GBP2 (0, 3, or 6 μg; **d**) or with siRNAs of *GBP2* (200 nM). Representative blots and quantified data are presented. Asterisks indicate statistically significant differences (*P < 0.05). Data are presented as mean ± SEM of three independent experiments. **f**, **g** The interaction between MCL-1 and BAK were evaluated in two independent GBP2 KO cell lines (**f**) or GBP2- or GBP1-stably overexpressing cell lines (**g**). Representative blots (left panel) and quantified data of bound BAK protein to MCL-1 (top on the right) and BAK protein level in total lysates (bottom on the right) are presented. The data are shown as mean ± SEM from three independent experiments. Asterisks indicate statistically significant differences (*P < 0.05; **P < 0.01; ***P < 0.005). Values denoted with different letters are statistically significant ones (*P < 0.05). **h** Changes in the levels of the BCL-2 family proteins after GBP2 knockdown (200 nM of siRNAs) or GBP2 overexpression (3 µg of plasmid) were determined by western blot analyses in HL-60 cells. Both representative blots and quantified data are presented. Data presented are the mean ± SEM of three independent experiments (*P < 0.05; **P < 0.01). **i** Changes in *BAK* mRNA levels were quantified by real-time PCR in K562 and HL-60 cells after transfection with siRNAs in GBP2 (200 nM) or GBP2 plasmid (3 µg) for 24 h. Data presented are mean ± SEM of three independent experiments performed in triplicate (*P < 0.05; **P < 0.01; ***P < 0.001).

Since the oligomerization of proapoptotic BCL-2 protein family members, BAK or BAX, induces MOMP [25], we further investigated whether these two proteins mediate GBP2-induced cell death using MEF cell lines established from *Bak*^*−/*−^, *Bax*^*−/−*^, and *Bax*^*−/−*^*Bak*^*−/−*^ double-knockout (KO) mice. Ectopic expression of GBP2 induced death of wild-type MEF cells to a similar extent as that of K562 cells. However, this GBP2-induced cytotoxic effect was completely abolished in both *Bak*^*−/−*^ and *Bax*^*−/−*^*Bak*^*−/*−^ cells, but not compromised in *Bax*^*−/*−^ cells (Fig. 3a). GBP1 also showed a *Bak*-dependent cell-killing effect, albeit with a weaker cell death activity compared with that of GBP2 (Fig. 3b). BAK-dependent and BAX-independent cell death effects of GBP2 were observed following the silencing of BAK or BAX in K562 cells (Fig. 3c), and similar results were observed in HL-60 cells (Supplementary Fig. 4).

The survival-promoting activity of MCL-1 involves its critical ability to sequester BAK by binding to its BH3 domain, which prevents BAK oligomerization at mitochondrial outer membranes [26]. Thus, we examined whether GBP2-induced BAK-dependent apoptosis involves the protein–protein interaction between GBP2 and MCL-1, which would consequently compromise the binding capacity of MCL-1 to sequester BAK. As shown in Fig. 3d, the physical interaction of MCL-1 with BAK was attenuated following GBP2 expression in a dose-dependent manner with a concomitant increase in MCL-1-binding to GBP2. Unlike BAK, MCL-1 did not interact with BAX in K562 cells (Fig. 3d). Similarly, GBP2 knockdown resulted in an enhanced complex formation between MCL-1 and BAK (Fig. 3e). We observed that GBP2 itself did not associate with either BAK or BAX (Supplementary Fig. 5). Moreover, we generated two independent GBP2 KO K562 cell lines using a CRISPR/Cas9-nickase-based system (Supplementary Fig. 6a), and sequencing and western blot analyses indicated that these cell lines would not express the full-length GBP2 (Fig. 3f and Supplementary Fig. 6b). Compared with WT K562 cells, elevated interaction of MCL-1 with BAK and downregulated levels of BAK protein were observed in these GBP2 KO cells generated (Fig. 3f). In sharp contrast, MCL-1/BAK interaction was significantly attenuated despite the prominent upregulation of BAK protein levels in the K562 cell lines stably overexpressing GBP2 or GBP1 (Fig. 3g). These results confirm our findings obtained using transiently GBP2-overexpressing cells (Fig. 3d) and GBP2-silenced cells using siRNAs (Fig. 3e).

We noticed that the modulation of GBP2 protein expression levels altered the expression of BAK (Fig. 3d–g). Moreover, the GBP2-induced regulation of BAK expression was confirmed at BAK protein level in HL-60 cells, while the levels of BAX and MCL-1 were not altered by GBP2 (Fig. 3h). These observations prompted us to determine the expression change of the *BAK* mRNA transcript following the modulation of GBP2 levels. Knockdown or overexpression of GBP2 significantly decreased or increased *BAK* mRNA level in K562 and HL-60 cells, respectively, as determined by qRT-PCR analysis (Fig. 3i). We further confirmed the downregulated *BAK* mRNA in the GBP2 KO cells (Supplementary Fig. 7). Therefore, these data collectively indicated that GBP2 dually regulates BAK by liberating BAK from the BAK-MCL-1 complex and upregulating BAK.

### PI3K/AKT pathway is a key mediator for the apoptosis-inducing function of GBP2

**Fig. 4:**
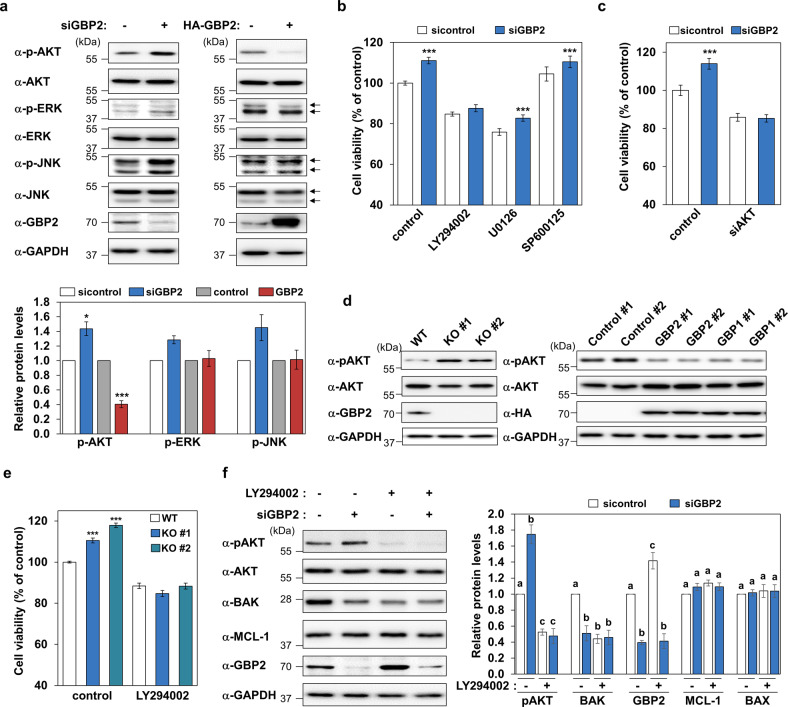
GBP2 inhibits AKT phosphoactivation, and the PI3K/AKT pathway is involved in GBP2-induced cell death. **a** The phosphorylation of different kinases was determined by western blot in *GBP2* overexpressed or silenced K562 cells. GAPDH was used as a loading control. Representative blots (upper panel) and quantitative data (lower panel) from three independent experiments are presented (*P < 0.05; ***P < 0.001). **b** K562 cells were transfected with siRNAs as control or GBP2, followed by incubation with DMSO (0.3%), LY294002 (30 μM), U0126 (20 μM), or SP600125 (20 μM) for 24 h. Data presented are mean ± SEM of three independent experiments performed in triplicate. Asterisks indicate statistically significant differences (***P < 0.001). **c** The effects of *AKT* silencing on the viability of K562 and GBP2-depleted cells were determined at 24 h after the knockdown of *AKT* using siRNAs (200 nM). Data presented are mean ± SEM of three independent experiments performed in triplicate. Asterisks indicate statistically significant differences (***P < 0.001). **d** Phosphoactivation of AKT was assessed by immunoblot analysis in the GBP2 KO and HA-GBP2- or HA-GBP1-stably overexpressing K562 cell lines. GAPDH was used as the loading control. **e** The effects of LY294002 (30 µM) on cell viability were determined in GBP2 KO cell lines after 24 h of incubation. Data were shown as mean ± SEM of three independent experiments performed in triplicate. Asterisks indicate statistically significant differences (***P < 0.001). **f** K562 cells were transfected with siGBP2 (200 nM) for 24 h, followed by treatment with LY294002 (30 μM) for an additional 24 h. Western blot analyses using indicated antibodies were performed. Representative blots (left panel) and quantitative data (right panel) from three independent experiments are presented. Different letters denote statistically significant differences (*P* < 0.05).

To identify kinases involved in the apoptotic function of GBP2, we evaluated the phosphoactivation of key kinases known to regulate cell survival and death after silencing or overexpression of GBP2. The phosphorylation of AKT at serine 473 was found to be dramatically increased or decreased in K562 cells by the knockdown or overexpression of *GBP2*, respectively (Fig. 4a). The phosphorylation of ERK2 at threonine 185 and tyrosine 187, and JNK at threonine 183 and tyrosine 185 tended to increase following *GBP2* silencing, but the opposite changes were not observed by GBP2 overexpression (Fig. 4a). LY294002, a PI3K inhibitor that blocks AKT phosphoactivation, completely prevented the cell survival-promoting activity induced by *GBP2* silencing, whereas inhibitors of other kinases, U0126 for ERK and SP600125 for JNK, failed to inhibit the survival-promoting effect of GBP2 depletion (Fig. 4b). The effectiveness of the inhibition of respective kinases by these antagonists at the used concentrations was confirmed by western blot analyses using phospho-specific antibodies (Supplementary Fig. 8a). To ascertain the crucial role of AKT in mediating GBP2-induced cell death function, the effect of *GBP2* silencing in AKT-knockdown cells was assessed, and we found that the survival-promotion effect induced by GBP2 depletion was also abolished by AKT silencing (Fig. 4c and Supplementary Fig. 8b). In addition, we also confirmed that AKT phosphorylation was increased or decreased in the GBP2 KO cell lines or the GBPs stably expressing cell lines, respectively (Fig. 4d). Consistent with the effects observed in the GBP2-knockdown cells following AKT inhibition (Fig. 4b, c), the promotion of cell survival activity induced by the knockout of GBP2 was prevented by inhibition of ATK using LY294002 (Fig. 4e). Furthermore, the increased AKT phosphorylation along with BAK downregulation upon *GBP2* knockdown was completely prevented by LY294002 treatment; BAX and MCL-1 levels were not drastically affected under the same conditions (Fig. 4f). Interestingly, LY294002 induced the upregulation of GBP2 (Fig. 4f).

### GBP2 mediates paclitaxel-induced cell death

**Fig. 5:**
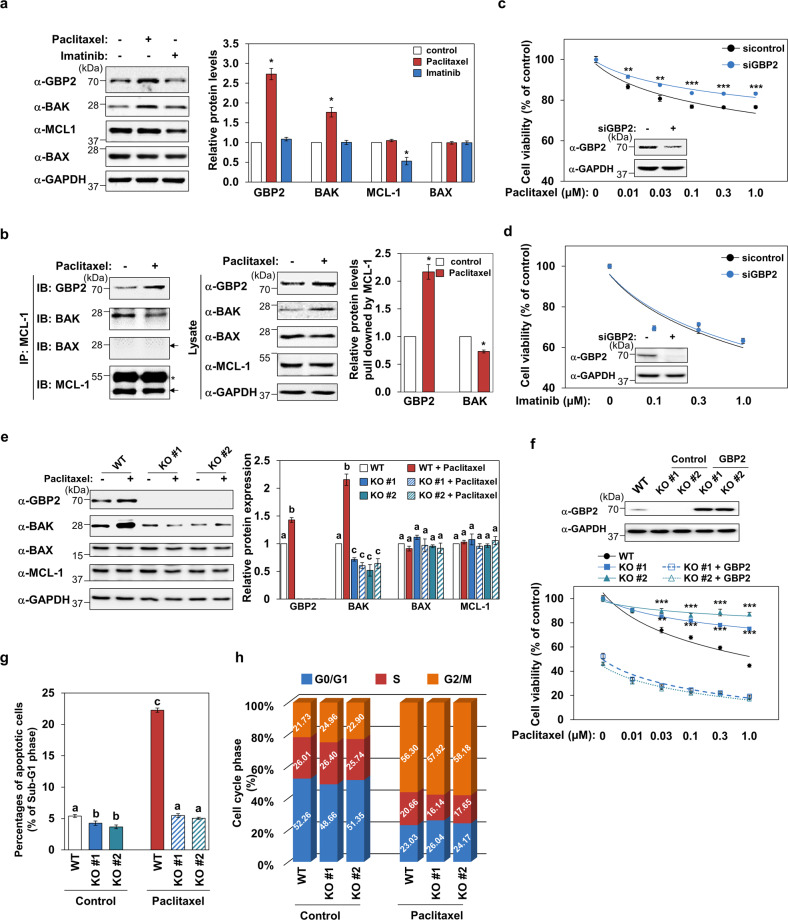
Paclitaxel upregulates GBP2, and GBP2 is a critical mediator for paclitaxel-induced cytotoxic effect on CML cells. **a** The changes in protein levels of GBP2, BCL-2 members, and AKT following the treatments with paclitaxel (1 μM) or imatinib (1 μM) for 24 h were evaluated by western blot analyses. Representative blots (left panel) and quantitative data (right panel) from three independent experiments are shown. Asterisks indicate statistically significant values (*P < 0.05). **b** The changes in the relative association of MCL-1 with GBP2 or BAK were assessed by immunoprecipitation followed by immune blot analyses of K562 cells treated with or without paclitaxel (1 µM) for 24 h. Representative (left) and quantified amounts (right) of GBP2 or BAK pulled downed by MCL-1 immunoprecipitants are presented from three independent experiments (*P < 0.05). **c**, **d** The effects of GBP2 silencing on paclitaxel- (**c**) or imatinib- (**d**) induced death of K562 cells were examined. Data presented are mean ± SEM of three independent experiments performed in triplicate (**P < 0.01; ***P < 0.001). **e** The protein level changes induced by paclitaxel (1 µM) were examined in GBP2 KO K562 cells by western blot analyses. Representative blots (left panel) and quantitative data (mean ± SEM; right panel) from three independent experiments are presented. Different letters indicate statistically significant differences (*P* < 0.05). **f** The changes in cell death activity induced by indicated concentrations of paclitaxel for 24 h were examined in GBP2 KO cell lines. In addition, the effects by knocking-in GBP2 were assessed by transfecting GBP2 KO K562 cells (1 × 10^4^) with empty vector or GBP2 expression plasmid (30 ng) for 24 h. Data were represented as mean ± SEM of three independent experiments performed in triplicate (**P < 0.01; ***P < 0.001). Cell lysates were subjected to western blot to confirm the expression of GBP2. Representative blots are shown, and GAPDH was used as a loading control. **g**, **h** The roles of GBP2 in paclitaxel-induced apoptosis (**g**) and cell cycle progression (**h**) in WT K562 cells and two independent GBP2 KO K562 cell lines were assessed using flow cytometry. Cells were exposed to DMSO (control) or paclitaxel (1 µM) for 24 h. The percentages of cells in the sub-G1 phase (**g**) and the G0/G1, S, and G2/M phases (**h**) are presented. Data are shown as mean ± SEM of three independent datasets. Different letters indicate statistically significant differences (*P* < 0.001).

To investigate the possible role of GBP2 against apoptosis induced by chemotherapeutics, K562 cells were treated with paclitaxel (Taxol^®^), a widely used anticancer drug, or imatinib (Greevec^®^), the first-line treatment for Philadelphia chromosome-positive CML. Changes in the expression level of GBP2, BAK, BAX, and MCL-1 were assessed. Immunoblot analysis results showed that paclitaxel dramatically increased GBP2 levels by accompanying BAK upregulation without affecting BAX and MCL-1 levels (Fig. 5a). In contrast, imatinib did not change the expression of GBP2, BAK, and BAX downregulated MCL-1 (Fig. 5a). In addition, we examined the changes in GBP2, BAK, and MCL-1 protein levels following exposure to the main chemotherapeutic agents used for the treatment of hematological malignancies, including doxorubicin, cytarabine, vincristine, etoposide, and interferon-γ. These chemotherapeutic agents also significantly upregulated GBP2 protein levels (Supplementary Fig. 9). However, BAK was not upregulated by any of the tested chemoagents, except paclitaxel (Supplementary Fig. 9). Thus, our data indicate that GBP2 upregulation is a shared feature of paclitaxel and other chemotherapeutics, but BAK upregulation is an effect unique to paclitaxel.

In addition, paclitaxel treatment in K562 cells increased MCL-1-bound GBP2, while the interaction of MCL-1 with BAK decreased as determined by immunoprecipitation (Fig. 5b), which would allow more freed apoptotic BAK available in cells. Paclitaxel-induced cytotoxic activity was partially inhibited in K562 cells with GBP2 knockdown (Fig. 5c). In contrast, the silencing of GBP2 did not significantly affect the cell death activity of imatinib (Fig. 5d).

Furthermore, we assessed paclitaxel-mediated regulation of GBP2 and BAK in GBP2 KO cells. BAK expression was significantly reduced in two independent GBP2 KO cell lines as determined by immunoblot analysis, and paclitaxel treatment in KO cells did not lead to the upregulation of GBP2 and BAK (Fig. 5e). Moreover, the paclitaxel-induced cell death effect was significantly decreased in these GBP2 KO cells (Fig. 5f). This reduction in paclitaxel-induced cell death observed in the GBP2 KO cells was effectively reversed by the knock-in of GBP2 in two independent GBP2 KO cell lines, thus confirming that GPB2 is a key mediator of paclitaxel-induced apoptosis in K562 leukemia cells (Fig. 5f).

To examine the mechanisms involved in paclitaxel-induced cytotoxicity, we analyzed apoptotic cells in the sub-G1 fraction. As presented in Fig. 5g, h, paclitaxel induced both apoptosis and cell cycle arrest in K562 (WT) cells. However, paclitaxel failed to induce apoptosis in GBP2 KO cells (Fig. 5g). In contrast, the cell cycle arrest-promoting activity of paclitaxel was not affected by GBP2 silencing (Fig. 5h). Thus, these data indicate that GBP2 is a key mediator of the apoptotic function of paclitaxel in K562 cells.

To elucidate the mechanisms involved in paclitaxel-induced GBP2 upregulation, we examined whether the inhibition of AKT phosphorylation induced by paclitaxel is associated with paclitaxel-induced GBP2 upregulation. As shown in Supplementary Fig. 10a, paclitaxel inhibited AKT phosphorylation in K562 cells. However, although LY294002-mediated inhibition of AKT phosphorylation increased GBP2 levels to some degree, paclitaxel was still able to dramatically increase GBP2 expression in AKT-inactivated cells (Supplementary Fig. 10a). These results suggest that paclitaxel-induced GBP2 upregulation is not dependent on its AKT inactivation activity.

In addition, we found that paclitaxel-induced upregulation of GBP2 protein does not involve the transcriptional regulation of *GBP2* mRNA (Supplementary Fig. 10b). Thus, we examined whether paclitaxel affected GBP2 protein stability. As shown in Supplementary Fig. 10c, the half-life of the GBP2 protein in untreated cells was ~16 h, whereas in paclitaxel-treated cells, GBP2 protein remained stable for at least 48 h (Supplementary Fig. 10c). When the cells were incubated with the proteasome inhibitor MG132, the stability of GBP2 protein in the presence and absence of paclitaxel was similar (Supplementary Fig. 10c). These data suggest that paclitaxel upregulates GBP2 levels by inhibiting the proteasomal degradation of the GBP2 protein.

### GBPs are underexpressed in leukemia patients and associated with a better prognosis

**Fig. 6:**
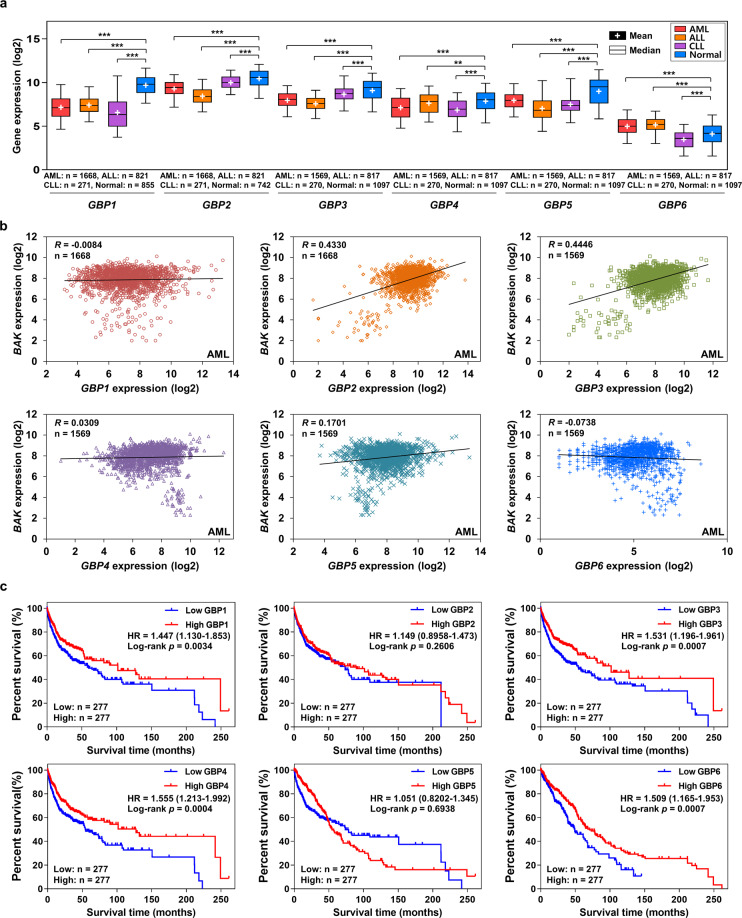
*GBP*s are downregulated in leukemia patients and associated with leukemia prognosis. **a** The mRNA expression (log2) data of the U133Plus2 platform were obtained from the gene expression across normal and tumor tissue (GENT2) portal (http://gent2.appex.kr/gent2/). The specific subtypes of leukemia-related datasets were selected for the gene expression study. Boxplot graphs of the expression levels of GBPs in normal tissue and specific leukemia subtypes (AML, ALL, and CLL) were constructed, and one-way ANOVA was used for statistical analysis. The box plot represents the lower, median, and upper quartiles, and the whiskers represent the 95% confidence interval of the mean. The sample sizes of each gene group were indicated in the figure. Asterisks indicate statistically significant differences (**P < 0.01; ***P < 0.001). **b** To study the correlation of *BAK* and *GBP*s, the Pearson correlation coefficient was used to assess the co-expression of *GBP2* and *BAK* genes in AML patients by analyzing the paired microarray data of AML patients extracted in (**a**). The estimated regression lines superimposed on the scatter plot of *BAK* with *GBPs* mRNAs, correlation coefficient (R), and the sample size are shown. **c** To study the prognostic value of the *GBP* gene family using their mRNA expression levels, the survival data of both leukemia and lymphoma patients were extracted from the subtype profiler of GENT2. Based on the median gene expression levels, patient data were divided into high expression and low expression groups. Overall survival curves were plotted for CN-AML, DLBCL, and CHL patients in relation to *GBP* expression levels (*n* = 277). The log-rank (Mantel–Cox) test was used to compare the survival distributions of patients with high and low expression of GBP genes. The hazard ratio (HR) together with the 95% CI and the *P* value of log-rank test are presented.

To examine clinical implications of the *GBP* gene family in leukemia, Affymetrix U133Plus2 microarray datasets comprising bone marrow and blood samples from normal and AML, ALL, and CLL patients were extracted and analyzed using the GENT2 portal [27]. The information of leukemia-related datasets used is described in supplementary Table 1. Due to the lack of available data, CML and *GBP7* could not be analyzed. The comparable mRNA levels of most *GBP* genes, (*GBP1–5*), except for *GBP6*, were significantly downregulated in the three types of leukemia (Fig. 6a). Comparative analyses of mRNA expression levels of *GBP* genes with *BAK* levels in tissues from AML patients revealed a strong positive correlation only between *GBP2* and *GBP3* and *BAK* (Fig. 6b). In addition, the relevance between the expression levels of GBPs and the prognosis of lymphoma and leukemia that include cytogenetically normal AML, diffuse large B-cell lymphoma (DLBCL), and classical Hodgkin lymphoma (CHL) were analyzed based on patient overall survival rate. Prognosis and mRNA expression data were extracted using the subtype profiler of GENT2. Due to the lack of prognosis data in most microarray datasets, only five datasets (GSE10846, GSE12417, GSE39133, GSE39134, and GSE53786) were analyzed for the survival analysis. The information of these five datasets is described in Supplementary Table 2. The survival data were divided by median expression level, and then the overall survival was analyzed by the log-rank (Mantel–Cox) test. *GBP1, GBP3, GBP4*, and *GBP6* were beneficial for overall survival, indicating that *GBP*s are favorable prognosis markers for tested leukemia and lymphoma types (Fig. 6c). Although *GBP2* and *GBP5* expression levels did not show significant benefit to leukemia and lymphoma patients, high expression of *GBP2* and *GBP5* showed trends of better prognosis in the long term (Fig. 6c).

## Discussion

The major known function of GBP proteins is mediating caspase 1- and 11-dependent pyroptosis during inflammasome activation upon various types of infections [28]. However, at present, their role in the regulation of apoptotic cell death has not been characterized. Here, we revealed that GBP1 and GBP2 are apoptosis-inducing molecules that activate caspases 3, 8, and 9, and further delineated their underlying mechanism for the first time. In particular, our finding elucidates the direct binding and regulation of GBPs with the BCL-2 family protein MCL-1 (Fig. 1), which introduces a new era of GBP proteins for their role in regulating cell survival and death. GBP2 inhibited the survival-promoting activity of MCL-1 in AML and CML cells by competitively binding to the BH3 domain of MCL-1, which consequently prevented BAK interaction with MCL-1, and thus the freed BAK readily oligomerized to induce MOMP (Figs. 2, 3d–g and Supplementary Fig. 3). Concurrently, GBP2 also upregulated apoptotic BAK expression through its inhibitory action on the PI3K-AKT pathway (Figs. 3i, 4 and Supplementary Fig. 7). To the best of our knowledge, the PI3K/AKT pathway-mediated BAK regulation has not previously reported in CML cells. However, in the case of T-cell acute lymphoblastic leukemia (T-ALL), one study showed that ZSTK-474, a pan PI3K p110 inhibitor, decreased BAK levels in a T-ALL cell line [29]. Our observations suggest that GBPs are dual regulators of BAK by stimulating both BAK liberation and upregulation. In addition, we demonstrated that compared with BAX for GBP1 and GBP2, BAK was indispensable to induce apoptotic cell death (Fig. 3a–c and Supplementary Fig. 4). Therefore, our present findings identified GBP1 and GBP2 as novel apoptosis-inducing proteins in myeloid leukemia cells, which regulate both MCL-1 and BAK; these are two critical members of the BCL-2 protein family presenting opposite functions to regulate the fate of cells by forming or disrupting the heterodimeric complex of MCL-1-BAK.

According to our data on the activation of caspase-8 and -9 (Fig. 2e), GBP1 and 2 likely induce apoptosis via both the intrinsic and extrinsic pathways. To clarify how caspase-8 is activated by GBPs, we evaluated the changes in the mRNA and protein levels of the death receptors FAS, TNFR1, and TRAILR1 in both GBP2 KO and GBP2-stably overexpressing cells. We found that the mRNA and protein levels of FAS in GBP2 KO and GBP2-stably overexpressing cells were significantly downregulated and upregulated, respectively (Supplementary Fig. 11a–d). In contrast, the levels of TNFR1 and TRAILR1 were not significantly affected by the modulation of the GBP2 expression levels in K562 cells (Supplementary Fig. 11a–d). Thus, these results suggest that GBP2-induced upregulation of FAS likely accounts for the GBP2-mediated activation of caspase-8.

At present, the roles of GBPs in oncogenic processes are not completely understood or are unclear because of the reported contradictory activities of GBPs. Higher expression of GBPs is associated with better prognosis in breast and colorectal carcinoma and skin cutaneous melanoma patients [18–20], but the opposite association was also reported for ovarian, glioblastoma, and esophageal and oral squamous cell carcinoma patients [30–33]. The molecular and cellular background for the context-dependent and heterogeneous roles of GBPs in cancers are not well understood yet and require future investigations. In this study, we revealed that *GBP1–5* are significantly downregulated in AML, ALL, or CLL patients compared with the non-leukemic population (Fig. 6a), and elevated levels of *GBP1, GBP3, GBP4*, and *GBP6* are associated with prolonged survival time in leukemia and lymphoma patients (Fig. 6c). This consistency in underexpressed GBP proteins in different types of leukemia and better prognosis with higher expression of *GBP*s in leukemia and lymphoma patients suggest the presence of a common anti-tumor activity of GBPs in leukemia and lymphoma. The relative comparison of *GBP2* transcript levels among 54 distinct human tissues analyzed [20] showed that whole blood expresses the most abundant levels of *GBP2*. This notion implies a particularly influential role of GBP2 in the hematological system among other GBP members. However, these bioinformatic data analyses also have limitations. Due to the limited available data for AML normal controls, which were defined as CD34^+^ hematopoietic progenitor cells, we performed more inclusive bioinformatic analyses that include leukemic cells in different stages of differentiation. Considering the well-characterized roles of GBPs in the host immune response, further studies are necessary to define how GBPs could act as leukemic suppressors in mechanisms involving either their intrinsic nature as apoptosis stimulators found in this study, their immunological activities associated with the cancer microenvironment, or both.

Paclitaxel (Taxol) is an antimicrotubule agent widely used for treating various types of solid tumors [34]. The known mode of action of paclitaxel is the inhibition of microtubule disassembly that induces mitotic arrest and apoptosis [35]. Although paclitaxel is currently not an established treatment option for treating CML, we found that paclitaxel induced apoptotic death of CML cells, and this activity was dependent on the presence of GBP2, as GBP2 KO cells were distinctively resistant to paclitaxel (Fig. 5f, g). This finding was in accordance with a recent study reporting that *GBP2* is downregulated in paclitaxel-resistant colorectal carcinoma cells and increases cell sensitivity to paclitaxel [36]. Of interest, paclitaxel dramatically upregulates GBP2 along with BAK, a process in which GBP2 was necessary for paclitaxel to increase BAK levels (Fig. 5a, e). Moreover, paclitaxel increased MCL-1 association to GBP2 while it decreased MCL-1-binding to BAK (Fig. 5b). These data revealed a previously unidentified mechanism of cytotoxic paclitaxel, where GBP2 acts as a critical mediator that upregulates and liberates BAK. Thus, these results suggest the possible application of paclitaxel as a combination therapy for CML treatment.

Elevated expression of MCL-1 has been observed in CML and AML patients and associated with poor clinical outcome and chemoresistance in leukemia cells [37, 38]. MCL-1 is essential for AML cell development and survival over other prosurvival BCL-2 members, including BCL-2, BCL-xL, or BCL-w [39]. In addition, upregulated MCL-1 is involved with BCR-ABL-mediated CML cell survival [40]. MCL-1 has been recognized as the major critical protein accounting for the resistance of leukemia cells against FDA-approved BH3 mimetic drugs, such as ABT-737 [38] that antagonizes BCL-2, BCL-xL, or BCL-w and venetoclax (ABT-199, the BCL-2 specific inhibitor) [41]. To overcome this resistance, efforts to develop MCL-1-selective BH3 mimetics have been recently made, and AMG176, S64315, and AZD5991 are currently approved in Phase I clinical trials for hematological malignancies [42, 43]. It is known that antiapoptotic BCL-2 family members have common and distinct interactions involving their BH3 domains [44]. Of note, a structural comparison revealed that the BH3 grooves of BCL-2 and BCL-xL were narrower than those of MCL-1 [45, 46]. Thus, we speculated that these structural differences in the BH3 groove could account for the specific association of GBP1/2 with MCL-1 and not with other antiapoptotic BCL-2 proteins.

Our findings of GBPs as dual regulators of MCL-1 and BAK via two different molecular mechanisms involving protein–protein interaction and transcriptional regulation, which allow synergistic amplification of apoptotic BAK activity, accentuate GBPs as new and attractive target molecules for leukemia therapeutics and perhaps for the treatment of other types of cancers.

## Materials and methods

### Plasmid constructions

Plasmids were generated by PCR amplification and cloned into vectors. The detailed information is shown in Supplementary Table 3. The Flag-tagged MCL-1 WT expression plasmid was constructed as previously described [47].

### Yeast two-hybrid system

The interaction of human GBP2 with MCL-1 was determined using a GAL4-based yeast two-hybrid system (Clontech, Palo Alto, CA, USA) as described previously [48].

### Immunoprecipitation and immunoblot analyses

Immunoprecipitation and immunoblotting were performed as previous description [49].

### Cell viability assay

Cell viability was measured via the CellTiter-Glo Assay (Promega, Madison, WI, USA) as described previously [50].

### Statistical analysis

The unpaired, two-tailed Student’s *t* test was used for comparisons with control using GraphPad PRISM (San Diego, CA, USA). Values marked with letters were analyzed by the Student–Newman–Keuls test using SAS version 9.2 (SAS Institute, Cary, NC, USA). Values marked with different letters indicate significantly different values. *P* < 0.05 was considered to be statistically significant.

Detailed descriptions of other methods are available in the Supplementary Methods.

## Supplementary information

Supplementary methods

Supplementary tables

Supplenmentary figures
