# Supplementary material for: Guanylate-binding proteins induce apoptosis of leukemia cells by regulating MCL-1 and BAK
Source: Oncogenesis. 2021 Jul 22;10(7):54. doi: 10.1038/s41389-021-00341-y (PMC8298518; doi:10.1038/s41389-021-00341-y)
Supplement: Supplementary file 1 — Supplementary methods [file 41389_2021_341_MOESM1_ESM.docx]

**Supplementary methods**

**Mammalian cell culture and transfection**

K562 (ATCC^®^ CCL-243™) and HL-60 cells (ATCC^®^ CCL-240™; ATCC, Manassas, VA, USA) were cultured in RPMI 1640 medium (Caisson, North Logan, UT, USA) supplemented with 10% fetal bovine serum (FBS) and 1% penicillin-streptomycin (PS). Dulbecco’s Modified Eagle Medium was used to culture 293T cells. Mouse embryo fibroblasts (MEFs) of *Bak^-/-^*, *Bax^-/-^*, and *Bax^-/-^Bak^-/-^* were generous gifts from Dr. CB Thompson (University of Pennsylvania, PA, USA) that were cultured using previous protocol^1^. Cells were grown in an incubator at 37°C with 5% CO_2_. K562, HL-60, and MEF cells were transfected using the Neon transfection system (Invitrogen, Carlsbad, CA, USA) according to the manufacturer’s instructions.

**Reagents and antibodies**

Z-VAD-FMK, U0126 (662005) was purchased from Calbiochem (San Diego, CA, USA). LY294002 (L9908), SP600125 (S5567), SB216763 (S3442) and paclitaxel (T7191) were purchased from Sigma-Aldrich (St. Louis, MO, USA). SAHB_B_ was a gift from Dr. HS Lim (POSTEC, Pohang, Korea). Imatinib was obtained from Santa Cruz Biotechnology (Santa Cruz, CA, USA). The anti-GBP2 (11854-1-AP) antibody was obtained from Proteintech (Chicago, IL, USA). The anti-GBP1 (sc-53857), anti-MCL-1 (sc-819), anti-BAX (sc-493), anti-cytochrome c (sc-7159), anti-β-actin (sc-47778), and anti-GAPDH (sc-25778) antibodies were purchased from Santa Cruz Biotechnology. The anti-FLAG M2 (F1804) and anti-HA (H6908) tag antibodies were purchased from Sigma-Aldrich. The anti-COX IV (A21347) and anti-BAK (AM03) antibodies were purchased from Invitrogen and Calbiochem, respectively. The anti-Caspase 3 (9662), anti-Caspase 8 (9746), anti-Caspase 9 (9508), anti-AKT (9272), anti-phospho-AKT (4058), anti-SAPK/JNK (9252), and anti-phospho-SAPK/JNK (9251) antibodies were purchased from Cell Signaling Technology (Danvers, MA, USA). The anti-ERK1/ERK2 (MAB1576) and anti-phospho-ERK1/ERK2 (AF1018) were obtained from R&D Systems (Minneapolis, MN, USA). The anti-His (34660) antibody was obtained from QIAGEN (Valencia, CA, USA). Alexa Fluor 546 goat anti-mouse IgG and Alexa Fluor 488 rabbit anti-goat IgG were purchased from Invitrogen.

**Immunocytochemistry**

Immunocytochemistry was carried out as previously described^3^. The anti-GBP2 antibody, anti-GBP1 and anti-MCL-1 were diluted at a ratio of 1: 100.

**Recombinant protein purification**

The purification of recombinant GBP2 and MCL-1 proteins from *Escherichia coli* strain BL21 (DE3) was performed as previously described^4^.

**RNA interference**

To silence GBP2, a mixture of the following four independent siRNAs targeting GBP2 were used: GBP2 #1 (5′-CCCAAUGAGCCUCAUUGAU-3′), GBP2 #2 (5′-GCCUGGGAGAUAUAGAGAA-3′), GBP2 #3 (5′-GGACAGUGAGAGAGAGGCCAUUGAA-3′), and GBP2 #4 (5′-CAU CAGAUUGUUGCAUGGCUUUACU-3′). The siRNA target sequence against MCL-1, BAK, and BAX were 5′-UAACACCAGUACGGACGGGUU-3′, 5′-GCUGGACAUUGGACUUCCU-3′, and 5′-AACCGACGCUAUGACUCAGAG-3′, respectively. siRNAs for AKT were designed as described previously^5^. The control scramble sequence used was 5′-CCUACGCCACCAAUUUCGU-3′.

**Measurement of the mitochondrial membrane permeabilization (MMP)**

MMP was measured using JC-1 (Invitrogen) and flow cytometric analysis as described previously^6^.

**Cytochrome *c* release**

Cytochrome *c* release was analyzed using a digitonin-based method that we described previously^7^.

**Generation of the cell lines stably overexpressing GBP1 or GBP2**

293T cells were used for lentivirus production by transfection of 6 µg of PMDLg/pRRE (#12251, Addgene, Watertown, MA, USA) and pRSV-Rev (#12253, Addgene), 4 µg of pMD2.G (#12259, addgene), and 8 µg of pLenti-puro empty vector (#39481, Addgene) or GBP1/2 encoded transfer vector for 48 h. Viral supernatant was collected for K562 cell infection in the presence of 10 µg/mL of polybrene (3.5 mL of lentivirus to 1 × 10^6^ cells in 7 ml media). After 48 h of infection, cells were selected with 2 µg/mL of puromycin for 30 days.

**Flow cytometry analysis of annexin V-positive cells**

To detect apoptotic cells, K562 cells (1 × 10^6^) were transfected with indicated plasmids or siRNAs. At 24 h post-transfection, cells were stained with the FITC-conjugated annexin V-FITC kit (BD Pharmingen, San Diego, CA, USA) according to the manufacturer’s instructions.

**Analysis of cell cycle phases by flow cytometry**

The cell cycle analysis was performed as previously described^8^. Cells were gated for the cell cycle analysis. For the detection of apoptotic cells in the sub-G1 phase, cells were analyzed without gating.

**Generation of** **CRISPR/Cas9-nickase-mediated *GBP2* KO cells**

GBP2 KO cells were generated as described before^3^. To generate GBP2 target CRISPR/Cas9-nickase plasmid, paired GBP2-targeted single guide RNA-1 and RNA-2 described in Supplementary Table 3 were used.

**RNA extraction and real-time PCR**

RNA extraction and real-time PCR were performed as described previously.^7^ The nucleotide sequences of primers (Bioneer) used for real-time PCR were as follows: GBP2-F (5′-TTTCCAGCATTTGTGTGGACT-3′), GBP2-R (5′-GGGAAGAACTTTCGGATGCAC-3′), MCL-1-F (5′-TGCTTCGGAAACTGGACATCA-3′), MCL-1-R (5′-TAGCCACAAAGGCACCAAAAG-3′), BAK-F (5′-CTGCAACCTAGCAGCACCAT-3′), BAK-R (5′-TGCTGGTAGACGTGTAGGGC-3′); GAPDH-F (5′-AGGGGCCATCCACAGTCTT-3′), and GAPDH-R (5′- AGCCAAAAGGGTCATCATCTCT-3′).

**Bioinformatics analyses**

To analyze mRNA expression levels of *GBPs* and their correlation with *BAK* expression, the mRNA expression (log2) data of the U133Plus2 platform were extracted from the gene expression database of normal and tumor tissues (GENT2) portal (<http://gent2.appex.kr/gent2/>)^9^. Subsequently, the specific subtypes of leukemia datasets (Supplementary Table 1) were selected for each gene expression analysis. The boxplot graphs of GBPs expression levels in normal tissue and leukemia subtypes were generated, and one-way ANOVA was used for the statistical analysis. The Pearson correlation coefficient was used to assess the correlation of *GBPs* and *BAK* levels. To study the prognostic value of *GBP* expression levels, the survival data of blood cancer patients was extracted from the subtype profiler of GENT2 (Supplementary Table 2). Based on the median of gene expression levels, patient data was divided into high and low expression groups. The overall survival was evaluated by a log rank (Mantel-Cox) test.

**Supplementary References**

1 Suh, D.-S., Park, S. E., Jin, H., Lee, K. & Bae, J. LRIG2 is a growth suppressor of Hec-1A and Ishikawa endometrial adenocarcinoma cells by regulating PI3K/AKT- and EGFR-mediated apoptosis and cell-cycle. *Oncogenesis* **7**, 3, doi:10.1038/s41389-017-0019-1 (2018).

2 Bae, J., Donigian, J. R. & Hsueh, A. J. Tankyrase 1 interacts with Mcl-1 proteins and inhibits their regulation of apoptosis. *The Journal of Biological Chemistry* **278**, 5195-5204, doi:10.1074/jbc.M201988200 (2003).

3 Jin, H. *et al.* FOXL2 directs DNA double-strand break repair pathways by differentially interacting with Ku. *Nature Communications* **11**, 2010, doi:10.1038/s41467-020-15748-1 (2020).

4 Park, M. *et al.* FOXL2 interacts with steroidogenic factor-1 (SF-1) and represses SF-1-induced CYP17 transcription in granulosa cells. *Molecular endocrinology (Baltimore, Md.)* **24**, 1024-1036, doi:10.1210/me.2009-0375 (2010).

5 Suh, D. S., Park, S. E., Jin, H., Lee, K. & Bae, J. LRIG2 is a growth suppressor of Hec-1A and Ishikawa endometrial adenocarcinoma cells by regulating PI3K/AKT- and EGFR-mediated apoptosis and cell-cycle. *Oncogenesis* **7**, 3, doi:10.1038/s41389-017-0019-1 (2018).

6 Kim, J. H. *et al.* HIP1R Interacts with a Member of Bcl-2 Family, BCL2L10, and Induces BAK-dependent Cell Death. *Cellular Physiology and Biochemistry* **23**, 043-052, doi:10.1159/000204088 (2009).

7 Kim, J. H. *et al.* MCL-1ES, a novel variant of MCL-1, associates with MCL-1L and induces mitochondrial cell death. *FEBS letters* **583**, 2758-2764, doi:10.1016/j.febslet.2009.08.006 (2009).

8 Suh, D. S. *et al.* Identification and Validation of Differential Phosphorylation Sites of the Nuclear FOXL2 Protein as Potential Novel Biomarkers for Adult-Type Granulosa Cell Tumors. *J Proteome Res* **14**, 2446-2456, doi:10.1021/pr501230b (2015).

9 Park, S.-J., Yoon, B.-H., Kim, S.-K. & Kim, S.-Y. GENT2: an updated gene expression database for normal and tumor tissues. *BMC Medical Genomics* **12**, 101, doi:10.1186/s12920-019-0514-7 (2019).
