# Supplementary material for: Guanylate-binding proteins induce apoptosis of leukemia cells by regulating MCL-1 and BAK
Source: Oncogenesis. 2021 Jul 22;10(7):54. doi: 10.1038/s41389-021-00341-y (PMC8298518; doi:10.1038/s41389-021-00341-y)
Supplement: Supplementary file 2 — Supplementary tables [file 41389_2021_341_MOESM2_ESM.docx]

**Supplementary Table 1. Microarray datasets extracted for gene expression analyses**

| **Gse-ID** | **Patient sample descriptions** |
| --- | --- |
| **Acute myeloid leukemia** | |
| GSE10358 | bone marrow (tumor) and matched skin biopsy samples (germline) from over 300 patients with de novo AML |
| GSE12326 | bone marrow (BM) and peripheral blood (PB) myeloblasts in acute myeloid leukemia (AML) |
| GSE12417 | 163 samples of bone marrow or peripheral blood mononuclear cells from adult patients with untreated acute myeloid leukemia. |
| GSE14479 | leukemic blasts from eight patients with acute myeloid leukemia (AML) carrying a CEBPA mutation and eight patients with AML without a CEBPA mutation but with silencing of CEBPA expression, and with nine samples of T-cell acute lymphoblastic leukemia (T-ALL) patients. |
| GSE15061 | 164 myelodysplastic syndrome (MDS), 202 AML and 69 non-leukemia bone marrow samples |
| GSE16015 | bone marrow samples, 631 AML patients with mutated/cytoplasmic NPM1 |
| GSE17061 | 35 AML FAB-M0 samples (AML), bone marrow aspirates or peripheral blood samples |
| GSE17855 | 237 bone marrow and peripheral blood samples from children with AML |
| GSE19577 | AML patients |
| GSE21261 | 96 bone marrow samples from patients with AML |
| GSE22056 | 98 bone marrow and peripheral blood samples in pediatric and adult acute myeloid leukemia |
| GSE6891 | 461 blood or bone marrow samples of acute myeloid leukemia patients |
| **Acute lymphoblastic leukemia** | |
| GSE10609 | T-ALL, viably frozen diagnostic bone marrow or peripheral blood samples from 92 pediatric patients with T-ALL |
| GSE10792 | bone marrow samples of childhood B-cell precursor ALL without known genetic aberrations |
| GSE10820 | lymphocytes from the peripheral blood samples of 11 patients with ALL |
| GSE11877 | children with high risk B-precursor ALL |
| GSE17459 | diagnostic bone marrow samples, Down's syndrome with acute lymphoblastic pediatric leukemia (DS-ALL) |
| GSE18239 | peripheral blood mononuclear cells (PBMCs) ALL |
| GSE19475 | bone marrow, infants (<1 year of age) diagnosed with ALL |
| GSE26713 | T-ALL, bone marrow control samples |
| GSE2842 | peripheral blood lymphoblasts purified at three time points (0 h, 6–8 h, 24 h after treatment initiation) from 13 children under therapy for ALL |
| GSE7440 | diagnostic marrow samples from 99 children with NCI high-risk B-precursor ALL treated on the protocol COG 1961 |
| **Chronic lymphoblastic leukemia** | |
| GSE10138 | 301 peripheral blood samples of chronic lymphocytic leukemia (CLL) |
| GSE12734 | B-cells from CLL patients |
| GSE14924 | T cells from patients with CLL |
| GSE14973 | CLL cells obtained from 14 patients |
| GSE15913 | peripheral blood samples of CLL patients |
| GSE21029 | peripheral blood (PB) vs bone marrow (BM) or lymph nodes (LN) derived CLL cells, CLL |
| GSE26526 | differential expression of the insulin receptor (INSR) in CLL |
| GSE9250 | CLL and subtypes of del13q14, peripheral blood |
| **Control blood** | |
| GSE11190 | hepatitis C virus (HCV), PBMCs isolated from blood |
| GSE4488 | whole blood |
| GSE7307 | bone marrow samples |
| GSE8052 | peripheral blood lymphocytes (PBL) |
| GSE14879 | anaplastic large cell lymphoma (ALCL), isolated from tonsils or blood of healthy donors |
| GSE5900 | bone marrow aspirates |
| GSE8023 | umbilical cord blood CD34+ cells |
| GSE6338 | 40 cases of peripheral T-cell lymphoma (PTCL) |
| GSE4588 | PBMC of patients with SLE, RA and healthy controls |
| GSE16059 | peripheral blood leucocytes in monozygotic twins discordant for chronic fatigue |
| GSE12408 | human lymphoblastoid cell lines (LCLs) |
| GSE14924 | peripheral blood T cells in acute myeloid leukemia (AML) |
| GSE15645 | peripheral blood mononuclear cells |
| GSE12195 | biopsies from 73 diffuse large B-cell lymphoma (DLBCL) patients |
| GSE13732 | Peripheral blood mononuclear cells |
| GSE13501 | peripheral blood |
| GSE13985 | white blood cells |
| GSE16363 | inguinal lymph node biopsies from 22 untreated HIV-1-infected subjects |
| GSE14642 | PBMCs were isolated from blood samples |
| GSE18781 | peripheral blood |
| GSE11504 | bone marrow from healthy humans |
| GSE16020 | polymorphonuclear leukocytes (PMNs) isolated from the blood of affected patients and healthy donors. |
| GSE19429 | bone marrow CD34+ cells |
| GSE16461 | peripheral blood mononuclear cell (PBMC) |
| GSE22501 | cord blood or peripheral blood |
| GSE25414 | white blood cell fraction |
| GSE26378 | whole blood |
| GSE26440 | whole blood |
| GSE26495 | peripheral blood cells |
| GSE27838 | non-expanded natural killer (NK) cells were isolated from PBMCs of healthy donors |

**Supplementary Table 2. GEO datasets extracted for survival analyses**

| **GSE-ID** | **Patient sample descriptions** |
| --- | --- |
| GSE10846 | diffuse large B cell lymphoma |
| GSE12417 | patients with cytogenetically normal acute myeloid leukemia (CN-AML) |
| GSE39133 | classical Hodgkin lymphoma |
| GSE39134 | classical Hodgkin lymphoma |
| GSE53786 | diffuse large B-cell lymphoma |

**Supplementary Table 3. Oligonucleotide sequences of cloning primers**

| **Plasmids** | **PCR primers** | **Vectors** | **Restriction enzyme sites** |
| --- | --- | --- | --- |
| HA-GBP2-WT | GBP2-F: 5’-GATGGATCCATGTATCCATATGATGTTCCAGATTATGCTATGGCTCCAGAGA-3’ | pcDNA3 vector  (Invitrogen, Carlsbad, CA, USA) | *BamH*I *and Xho*I  (Enzynomics, Seoul, Korea) |
|  | GBP2-R: 5′-GATCTCGAGTTAGAGTATGTTACATAT-3’ |  |  |
| HA-GBP2-ΔC | GBP2-F: 5’-GATGGATCCATGTATCCATATGATGTTCCAGATTATGCTATGGCTCCAGAGA-3’ | pcDNA3 vector  (Invitrogen) | *BamH*I *and Xho*I  (Enzynomics) |
|  | GBP2-ΔC-R: 5′-GCACTCGAGTTAGCAGGGTAGATCCCCA-3′ |  |  |
| HA-GBP2-ΔN | GBP2-ΔN-F: 5′-GATGGATCCATGTATCCATATGATGTTCCAGATTATGCTATGGAGAACGCAGT-3′ | pcDNA3 vector  (Invitrogen) | *BamH*I *and Xho*I  (Enzynomics) |
|  | GBP2-R: 5′-GATCTCGAGTTAGAGTATGTTACATAT-3’ |  |  |
| HA-GBP1-WT | GBP1-F: 5’-AGTGGATCCATGTACCCATACGATGTTCCAGATTACGCTATGGCATCAGAGATCCACAT-3’ | pcDNA3 vector  (Invitrogen) | *BamH*I *and EcoR*I (Enzynomics) |
|  | GBP1-R: 5′-GCAGAATTCTTACAGGCTCTCTAGACGAGGCC-3’ |  |  |
| HA-GBP1-ΔC | GBP1-F: 5’-GATGGATCCATGTATCCATATGATGTTCCAGATTATGCTATGGCTCCAGAGA-3’ | pcDNA3 vector  (Invitrogen) | *BamH*I *and EcoR*I  (Enzynomics) |
|  | GBP1-ΔC-R: 5′-GCAGAATTCTTACAGGCTCTCTAGACGAGGCC-3′ |  |  |
| HA-GBP1-ΔN | GBP1-ΔN-F: 5’-AGTGGATCCATGTACCCATACGATGTTCCAGATTACGCTATGGTGCTGACCTACGTCAATGC-3’ | pcDNA3 vector  (Invitrogen) | *BamH*I *and EcoR*I  (Enzynomics) |
|  | GBP1-R: 5′-GCAGAATTCTTACAGGCTCTCTAGACGAGGCC-3’ |  |  |
| MCL-1 BH3M | MCL1 BH3 M-R: 5′-GAGCACCAACGCGACGTGACGACGACGACGACGACGACGCCAGAGGTCGCGGAAGGACGA-3′) | pcDNA3 vector  (Invitrogen) | *EcoR*I *and Xba*I  (Enzynomics) |
|  | MCL1 BH3M-F: (5′-GCCACCAGCAGGAAGGCGCTGGAGACC-3′ |  |  |
| FLAG-BCL-2 | BCL2-F: 5′-CTAGAATTCATGGACTACAAAGACGACGACGACAAATCCGCGCACG-3′ | pcDNA3 vector  (Invitrogen) | *EcoR*I *and Xho*I  (Enzynomics) |
|  | BCL2-R: 5′-CTACTCGAGTCACTTGTGGCCCAGATA-3′ |  |  |
| FLAG-BCL-xL | BCL-xL-F: 5′-AGTGAATTCATGGACTACAAAGACGACGACGACAAATCCCAGAGTAACC-3’ | pcDNA3 vector  (Invitrogen) | *EcoR*I *and Xho*I  (Enzynomics) |
|  | BCL-xL-R: 5′-CTACTCGAGTCATTTCCGACTGAAGAG-3′ |  |  |
| FLAG-BCL2A1 | BCL2A1-F: 5´-TTAGAATTCATGGACTACAAAGACGACGACGACAAATCCACAGACTGTGAA-3′ | pcDNA3 vector  (Invitrogen) | *EcoR*I *and Xho*I  (Enzynomics) |
|  | BCL2A1-R: 5´- CTACTCGAGTCAACAGTATTGCTTCAG-3′ |  |  |
| GBP2 bacterial expression plasmid | pET-GBP2-F: 5’-CTAGGATCCATGGCTCCAGAGATCAACTTGC-3’ | pET28a  (Novagen, San Diego, CA, USA) | *BamH*I *and Xho*I  (Enzynomics) |
|  | pET-GBP2-R: 5’-GCGCTCGAGTTAGAGTATGTTACATATTGGCTCC-3′ |  |  |
| GBP1 encoded plenti.puro plasmid | HA-GBP1-F: 5’-AGTGGATCCATGTACCCATACGATGTTCCAGATTACGCTATGGCATCAGAGATCCACAT-3’ | pLenti-puro  (Addgene,Cambridge, MA, USA) | *BamH*I *and EcoR*I (Enzynomics) |
|  | HA-GBP1-R: 5’-GCAGAATTCTTAGCTTATGGTACATGCCT-3’ |  |  |
| GBP2 encoded plenti.puro plasmid | HA-GBP2-F: 5’-AGTGGATCCATGTACCCATACGATGTTCCAGATTACGCTATGGCTCCAGAGATCAACTT-3’ | pLenti-puro  (Addgene) | *BamH*I *and Xho*I  (Enzynomics) |
|  | HA-GBP2-R: 5’-AGACTCGAGTTAGAGTATGTTACATATTGGCTCC-3’ |  |  |
| GBP2-targeted single guide RNA-1 | sgRNA-1-F: 5′- CACCGGTAAAGAGACGGTAACCTCC-3′ | Cas9 (D10A) mutagenized pSpCas9n(BB)-2A-GFP  (Addgene) | *Bbs*I  (Thermo Fisher Scientific, Waltham, MA, USA) |
|  | sgRNA-1-R: 5′-AAACGGAGGTTACCGTCTCTTTACC-3′ |  |  |
| GBP2-targeted single guide RNA-2 | sgRNA-2-F: 5′-CACCGGCTGAAGAATAAGTACTACC-3′ | Cas9 (D10A) mutagenized pSpCas9n(BB)-2A-GFP  (Addgene) | *Bbs*I  (Thermo Fisher Scientific) |
|  | sg-RNA-2-R: 5′- AAACGGTAGTACTTATTCTTCAGCC-3′ |  |  |
