# Supplementary material for: Guanylate-binding proteins induce apoptosis of leukemia cells by regulating MCL-1 and BAK
Source: Oncogenesis. 2021 Jul 22;10(7):54. doi: 10.1038/s41389-021-00341-y (PMC8298518; doi:10.1038/s41389-021-00341-y)
Supplement: Supplementary file 3 — Supplenmentary figures [file 41389_2021_341_MOESM3_ESM.pptx]

## Slide 1
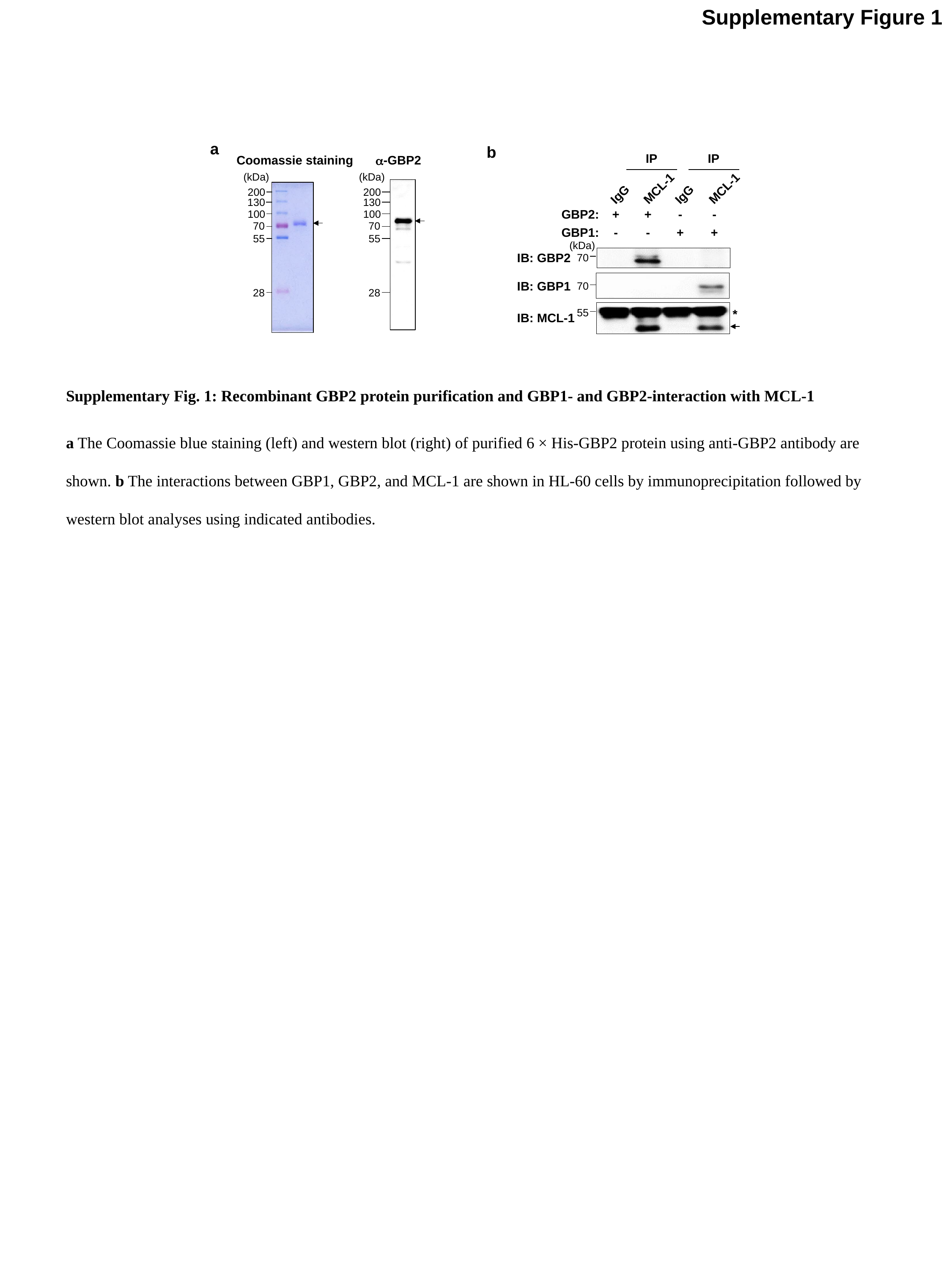

Supplementary Figure 1
a
b
IP
IP
MCL-1
MCL-1
IgG
IgG
GBP2:
+
+
-
-
GBP1:
-
-
+
+
(kDa)
IB: GBP2
70
IB: GBP1
70
55
*
IB: MCL-1
Coomassie staining
α-GBP2
(kDa)
200
130
100
70
55
28
(kDa)
200
130
100
70
55
28
Supplementary Fig. 1: Recombinant GBP2 protein purification and GBP1- and GBP2-interaction with MCL-1
a The Coomassie blue staining (left) and western blot (right) of purified 6 × His-GBP2 protein using anti-GBP2 antibody are shown. b The interactions between GBP1, GBP2, and MCL-1 are shown in HL-60 cells by immunoprecipitation followed by western blot analyses using indicated antibodies.

## Slide 2
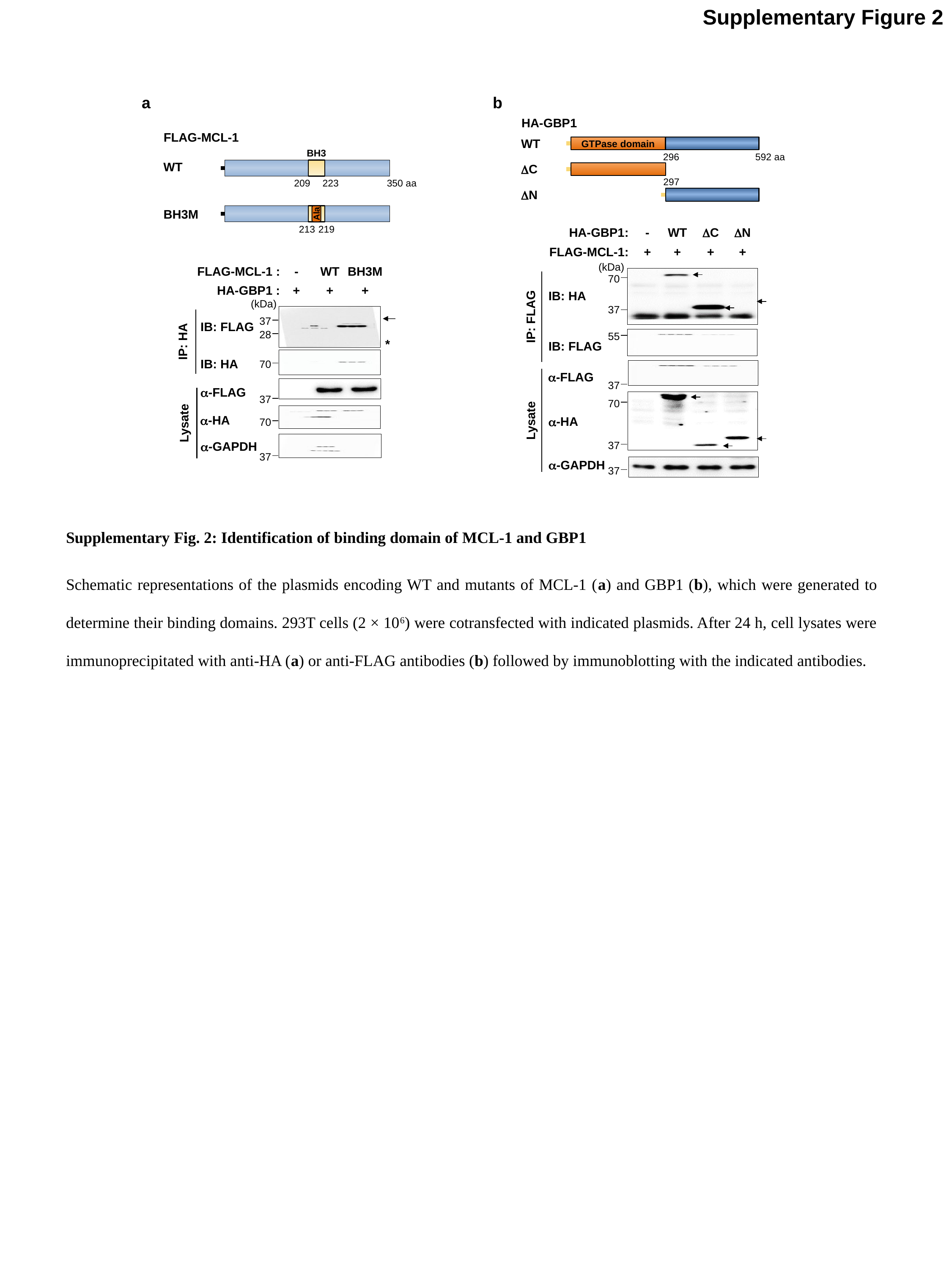

Supplementary Figure 2
a
b
HA-GBP1
FLAG-MCL-1
WT
GTPase domain
BH3
296
592 aa
WT
ΔC
297
209
223
350 aa
ΔN
BH3M
Ala
213
219
HA-GBP1:
-
WT
ΔC
ΔN
FLAG-MCL-1:
+
+
+
+
(kDa)
FLAG-MCL-1 :
-
WT
BH3M
70
HA-GBP1 :
+
+
+
IB: HA
(kDa)
37
IP: FLAG
37
IB: FLAG
28
55
IP: HA
*
IB: FLAG
IB: HA
70
α-FLAG
37
α-FLAG
37
70
α-HA
Lysate
α-HA
70
Lysate
37
α-GAPDH
37
α-GAPDH
37
Supplementary Fig. 2: Identification of binding domain of MCL-1 and GBP1
Schematic representations of the plasmids encoding WT and mutants of MCL-1 (a) and GBP1 (b), which were generated to determine their binding domains. 293T cells (2 × 10^6^) were cotransfected with indicated plasmids. After 24 h, cell lysates were immunoprecipitated with anti-HA (a) or anti-FLAG antibodies (b) followed by immunoblotting with the indicated antibodies.

## Slide 3
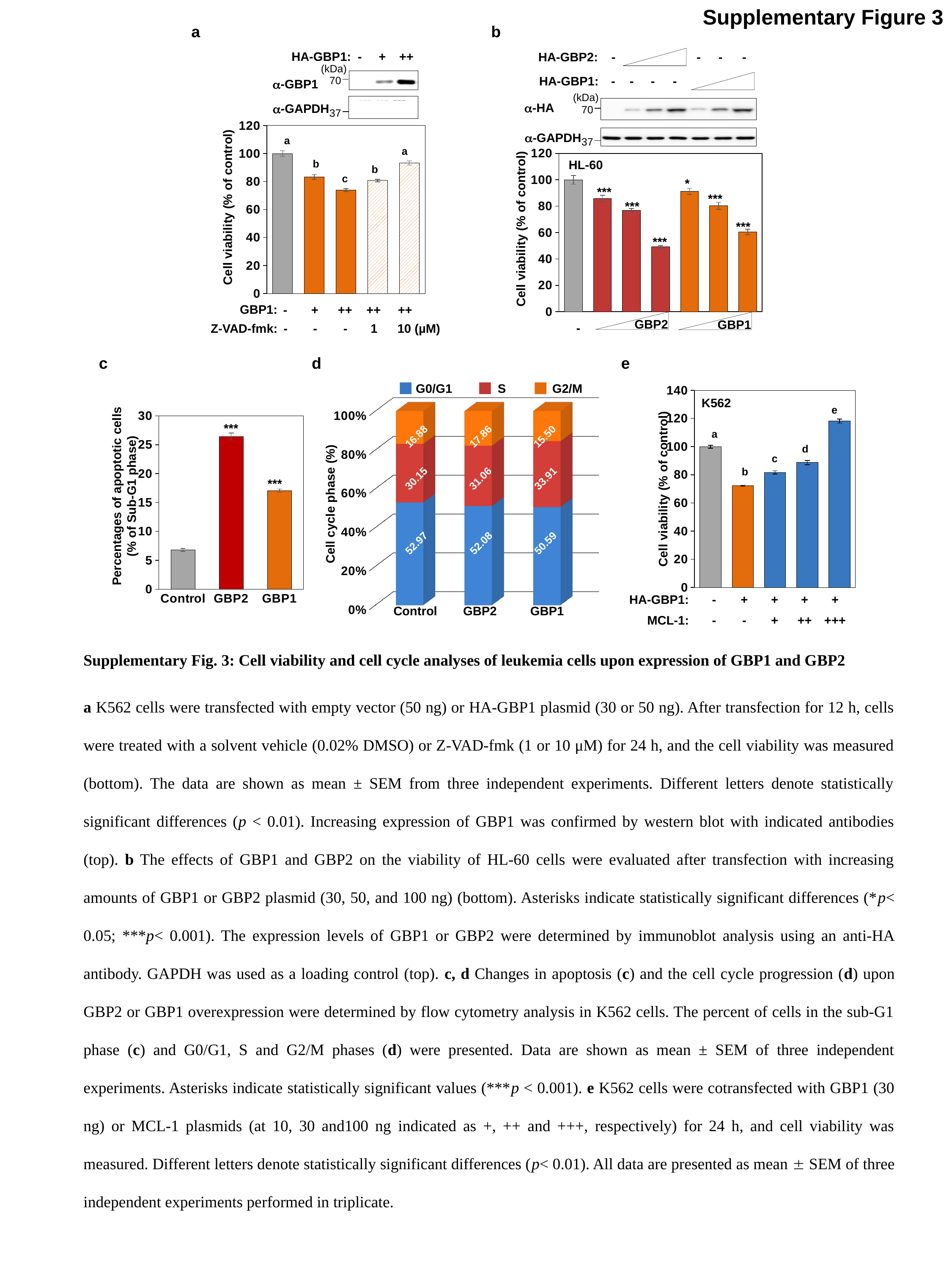

Supplementary Figure 3
a
b
HA-GBP1:
-
+
++
(kDa)
70
α-GBP1
α-GAPDH
37
### Chart
| Category | |
|---|---|a
a
b
b
c
Cell viability (% of control)
GBP1:
-
+
++
++
++
Z-VAD-fmk:
-
-
-
1
10 (µM)
HA-GBP2:
-
-
-
-
HA-GBP1:
-
-
-
-
(kDa)
α-HA
70
α-GAPDH
37
### Chart
| Category | |
|---|---|HL-60
*
***
***
***
***
Cell viability (% of control)
***
GBP2
GBP1
-
c
d
e
G0/G1
S
G2/M
[unsupported chart]
16.88
17.86
15.50
30.15
31.06
33.91
Cell cycle phase (%)
52.97
52.08
50.59
Control
GBP2
GBP1
### Chart
| Category | |
|---|---|K562
Cell viability (% of control)
e
a
d
c
b
HA-GBP1:
-
+
+
+
+
MCL-1:
-
-
+
++
+++
### Chart
| Category | |
|---|---|
| Control | 6.8 |
| GBP2 | 26.433333333333334 |
| GBP1 | 17.033333333333335 |Percentages of apoptotic cells (% of Sub-G1 phase)
***
***
Supplementary Fig. 3: Cell viability and cell cycle analyses of leukemia cells upon expression of GBP1 and GBP2
a K562 cells were transfected with empty vector (50 ng) or HA-GBP1 plasmid (30 or 50 ng). After transfection for 12 h, cells were treated with a solvent vehicle (0.02% DMSO) or Z-VAD-fmk (1 or 10 μΜ) for 24 h, and the cell viability was measured (bottom). The data are shown as mean ± SEM from three independent experiments. Different letters denote statistically significant differences (p < 0.01). Increasing expression of GBP1 was confirmed by western blot with indicated antibodies (top). b The effects of GBP1 and GBP2 on the viability of HL-60 cells were evaluated after transfection with increasing amounts of GBP1 or GBP2 plasmid (30, 50, and 100 ng) (bottom). Asterisks indicate statistically significant differences (*p< 0.05; ***p< 0.001). The expression levels of GBP1 or GBP2 were determined by immunoblot analysis using an anti-HA antibody. GAPDH was used as a loading control (top). c, d Changes in apoptosis (c) and the cell cycle progression (d) upon GBP2 or GBP1 overexpression were determined by flow cytometry analysis in K562 cells. The percent of cells in the sub-G1 phase (c) and G0/G1, S and G2/M phases (d) were presented. Data are shown as mean ± SEM of three independent experiments. Asterisks indicate statistically significant values (***p < 0.001). e K562 cells were cotransfected with GBP1 (30 ng) or MCL-1 plasmids (at 10, 30 and100 ng indicated as +, ++ and +++, respectively) for 24 h, and cell viability was measured. Different letters denote statistically significant differences (p< 0.01). All data are presented as mean ± SEM of three independent experiments performed in triplicate.

## Slide 4
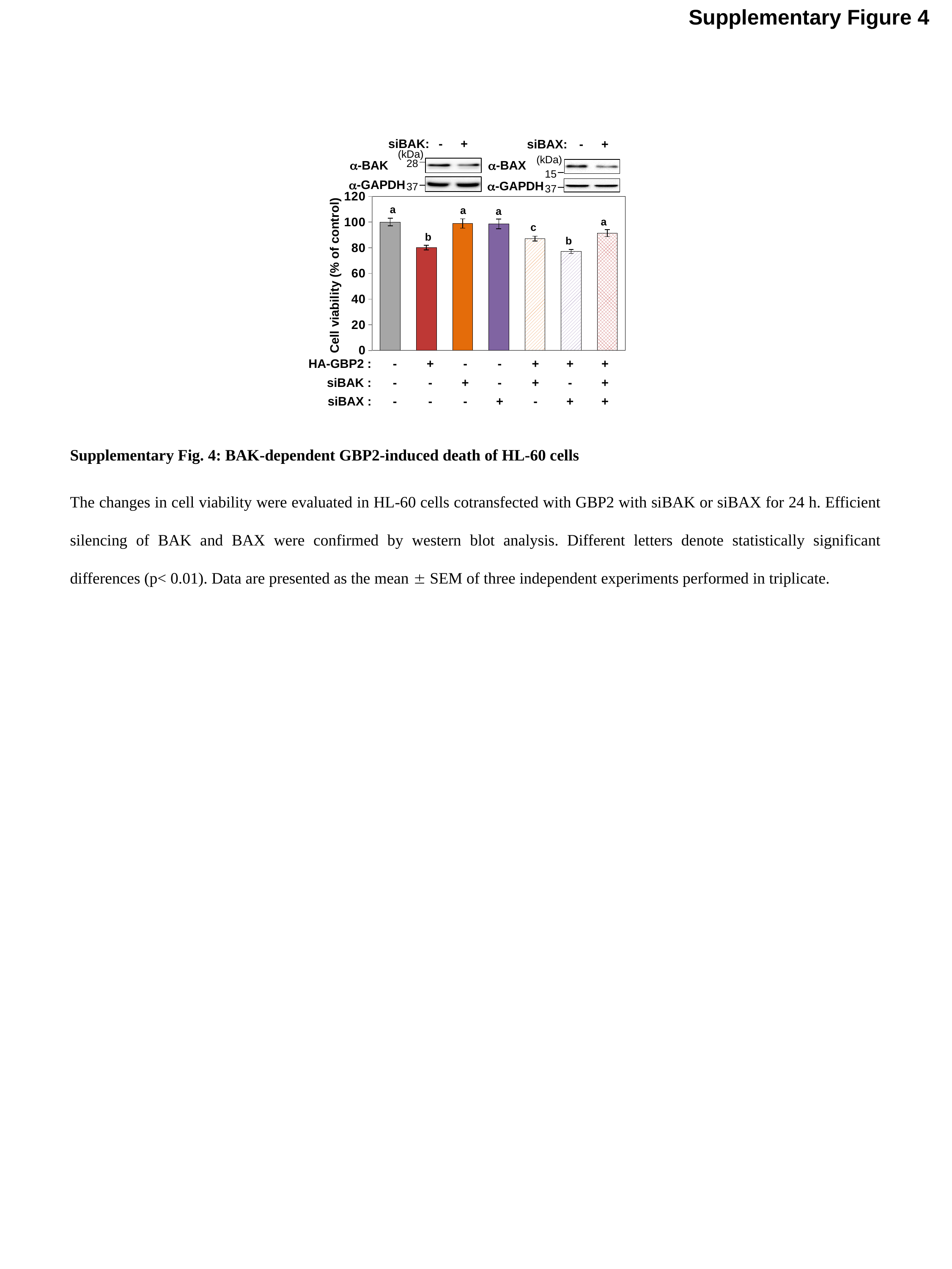

Supplementary Figure 4
siBAK:
-
+
siBAX:
-
+
(kDa)
(kDa)
28
α-BAK
α-BAX
15
α-GAPDH
α-GAPDH
37
37
### Chart
| Category | |
|---|---|a
a
a
a
c
b
b
Cell viability (% of control)
HA-GBP2 :
-
+
-
-
+
+
+
siBAK :
-
-
+
-
+
-
+
siBAX :
-
-
-
+
-
+
+
Supplementary Fig. 4: BAK-dependent GBP2-induced death of HL-60 cells
The changes in cell viability were evaluated in HL-60 cells cotransfected with GBP2 with siBAK or siBAX for 24 h. Efficient silencing of BAK and BAX were confirmed by western blot analysis. Different letters denote statistically significant differences (p< 0.01). Data are presented as the mean ± SEM of three independent experiments performed in triplicate.

## Slide 5
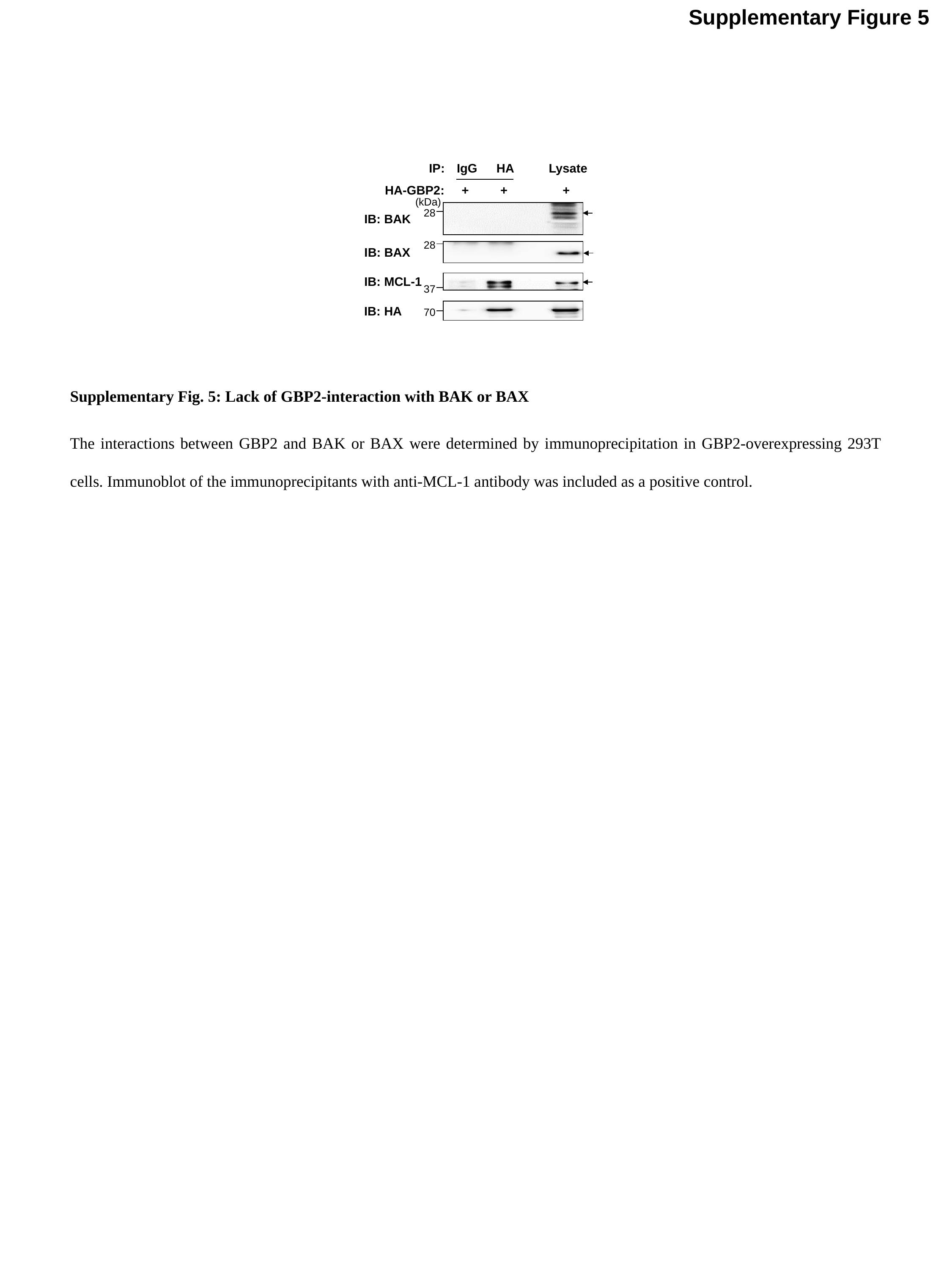

Supplementary Figure 5
IP:
IgG
HA
Lysate
HA-GBP2:
+
+
+
(kDa)
28
IB: BAK
28
IB: BAX
IB: MCL-1
37
IB: HA
70
Supplementary Fig. 5: Lack of GBP2-interaction with BAK or BAX
The interactions between GBP2 and BAK or BAX were determined by immunoprecipitation in GBP2-overexpressing 293T cells. Immunoblot of the immunoprecipitants with anti-MCL-1 antibody was included as a positive control.

## Slide 6
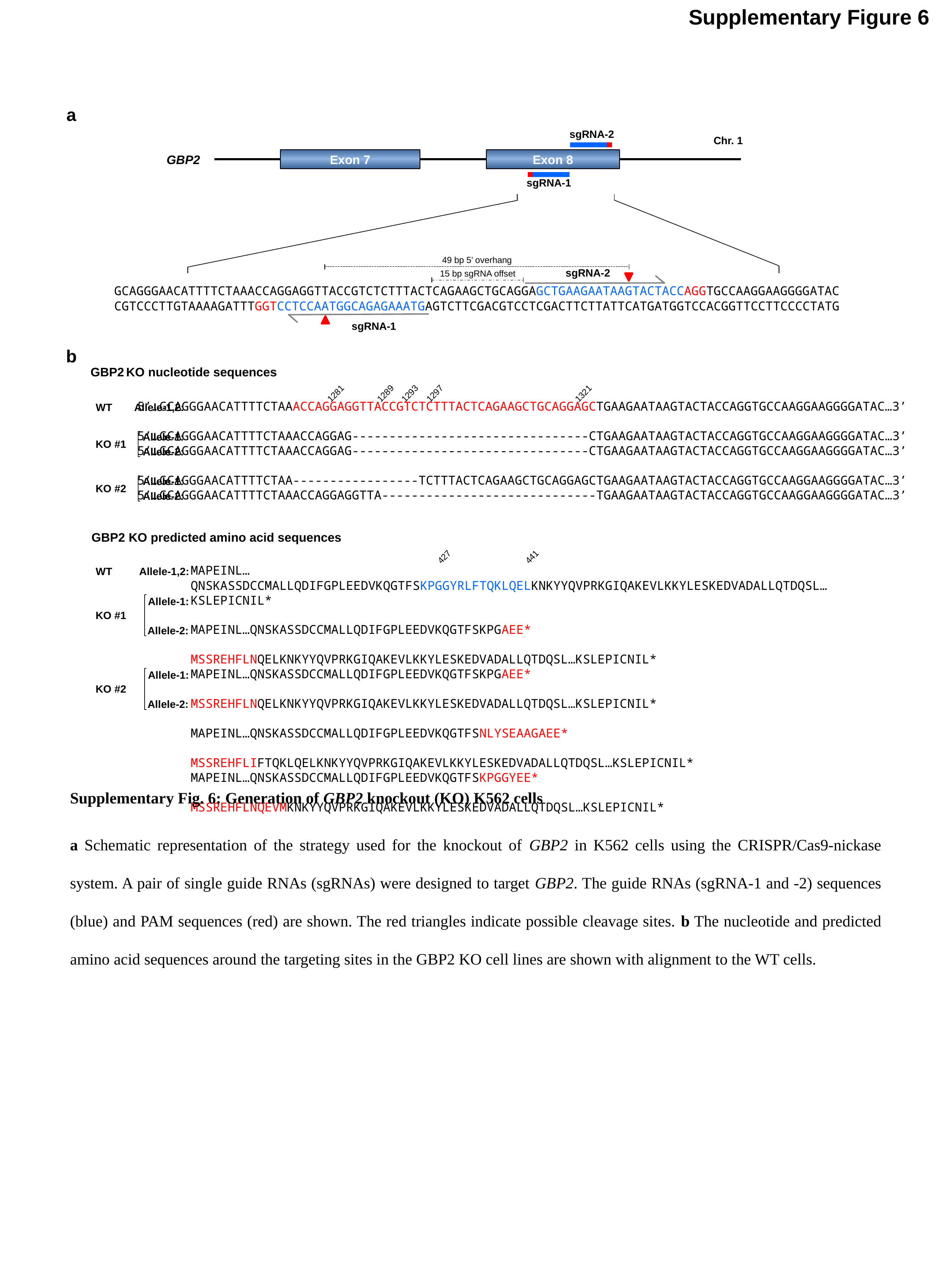

Supplementary Figure 6
a
sgRNA-2
Chr. 1
GBP2
Exon 7
Exon 8
sgRNA-1
49 bp 5’ overhang
sgRNA-2
15 bp sgRNA offset
GCAGGGAACATTTTCTAAACCAGGAGGTTACCGTCTCTTTACTCAGAAGCTGCAGGAGCTGAAGAATAAGTACTACCAGGTGCCAAGGAAGGGGATAC
CGTCCCTTGTAAAAGATTTGGTCCTCCAATGGCAGAGAAATGAGTCTTCGACGTCCTCGACTTCTTATTCATGATGGTCCACGGTTCCTTCCCCTATG
sgRNA-1
b
GBP2 KO nucleotide sequences
1281
1289
1293
1297
1321
5’…GCAGGGAACATTTTCTAAACCAGGAGGTTACCGTCTCTTTACTCAGAAGCTGCAGGAGCTGAAGAATAAGTACTACCAGGTGCCAAGGAAGGGGATAC…3’
5’…GCAGGGAACATTTTCTAAACCAGGAG--------------------------------CTGAAGAATAAGTACTACCAGGTGCCAAGGAAGGGGATAC…3’
5’…GCAGGGAACATTTTCTAAACCAGGAG--------------------------------CTGAAGAATAAGTACTACCAGGTGCCAAGGAAGGGGATAC…3’
5’…GCAGGGAACATTTTCTAA-----------------TCTTTACTCAGAAGCTGCAGGAGCTGAAGAATAAGTACTACCAGGTGCCAAGGAAGGGGATAC…3’
5’…GCAGGGAACATTTTCTAAACCAGGAGGTTA-----------------------------TGAAGAATAAGTACTACCAGGTGCCAAGGAAGGGGATAC…3’
WT
Allele-1,2:
Allele-1:
Allele-2:
KO #1
Allele-1:
Allele-2:
KO #2
GBP2 KO predicted amino acid sequences
427
441
MAPEINL…QNSKASSDCCMALLQDIFGPLEEDVKQGTFSKPGGYRLFTQKLQELKNKYYQVPRKGIQAKEVLKKYLESKEDVADALLQTDQSL…KSLEPICNIL*
MAPEINL…QNSKASSDCCMALLQDIFGPLEEDVKQGTFSKPGAEE*
 MSSREHFLNQELKNKYYQVPRKGIQAKEVLKKYLESKEDVADALLQTDQSL…KSLEPICNIL*
MAPEINL…QNSKASSDCCMALLQDIFGPLEEDVKQGTFSKPGAEE*
 MSSREHFLNQELKNKYYQVPRKGIQAKEVLKKYLESKEDVADALLQTDQSL…KSLEPICNIL*
MAPEINL…QNSKASSDCCMALLQDIFGPLEEDVKQGTFSNLYSEAAGAEE*
 MSSREHFLIFTQKLQELKNKYYQVPRKGIQAKEVLKKYLESKEDVADALLQTDQSL…KSLEPICNIL*
MAPEINL…QNSKASSDCCMALLQDIFGPLEEDVKQGTFSKPGGYEE*
 MSSREHFLNQEVMKNKYYQVPRKGIQAKEVLKKYLESKEDVADALLQTDQSL…KSLEPICNIL*
WT
Allele-1,2:
Allele-1:
Allele-2:
KO #1
Allele-1:
Allele-2:
KO #2
Supplementary Fig. 6: Generation of GBP2 knockout (KO) K562 cells
a Schematic representation of the strategy used for the knockout of GBP2 in K562 cells using the CRISPR/Cas9-nickase system. A pair of single guide RNAs (sgRNAs) were designed to target GBP2. The guide RNAs (sgRNA-1 and -2) sequences (blue) and PAM sequences (red) are shown. The red triangles indicate possible cleavage sites. b The nucleotide and predicted amino acid sequences around the targeting sites in the GBP2 KO cell lines are shown with alignment to the WT cells.

## Slide 7
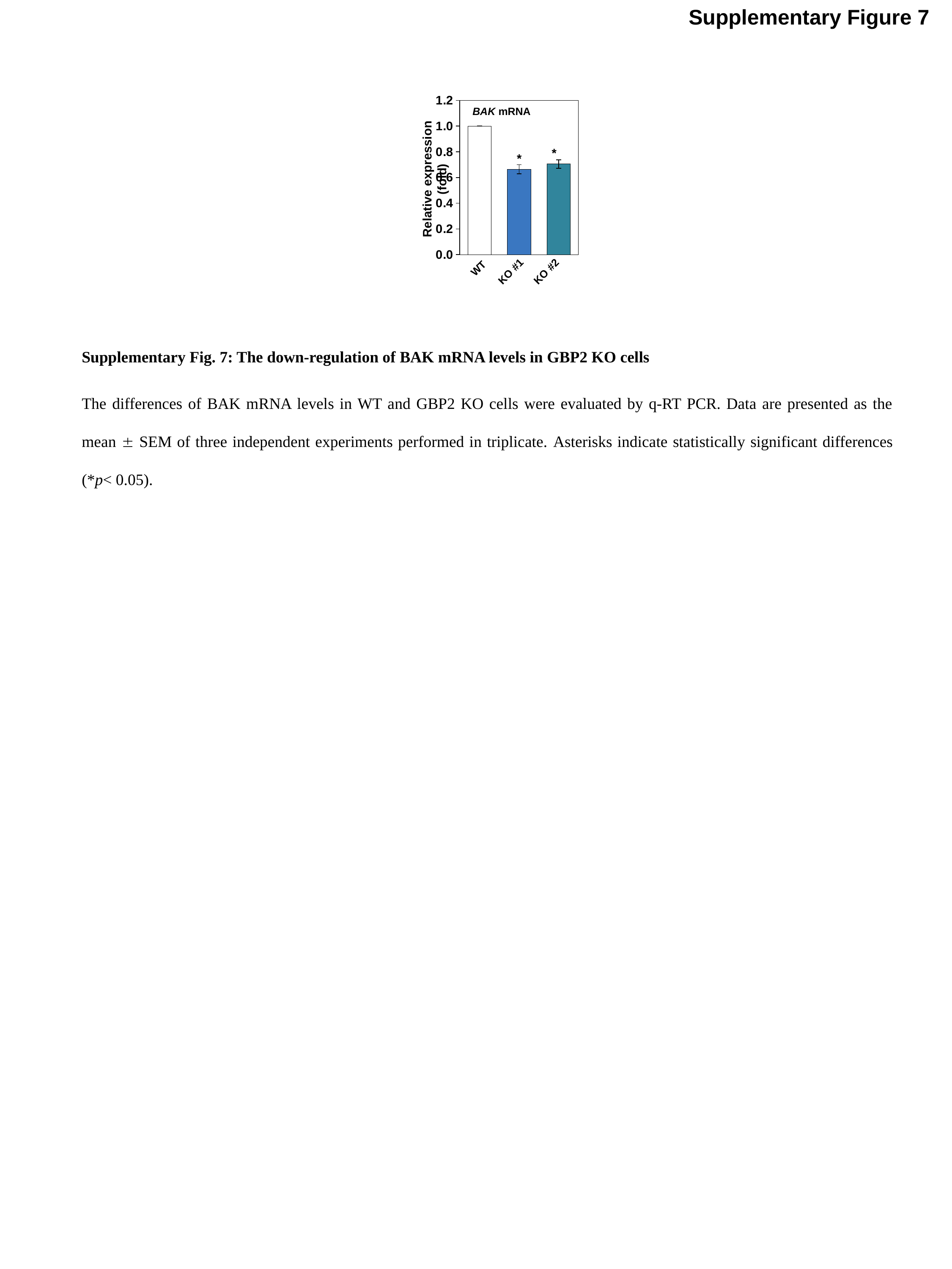

Supplementary Figure 7
### Chart
| Category | |
|---|---|BAK mRNA
*
*
Relative expression (fold)
WT
KO #1
KO #2
Supplementary Fig. 7: The down-regulation of BAK mRNA levels in GBP2 KO cells
The differences of BAK mRNA levels in WT and GBP2 KO cells were evaluated by q-RT PCR. Data are presented as the mean ± SEM of three independent experiments performed in triplicate. Asterisks indicate statistically significant differences (*p< 0.05).

## Slide 8
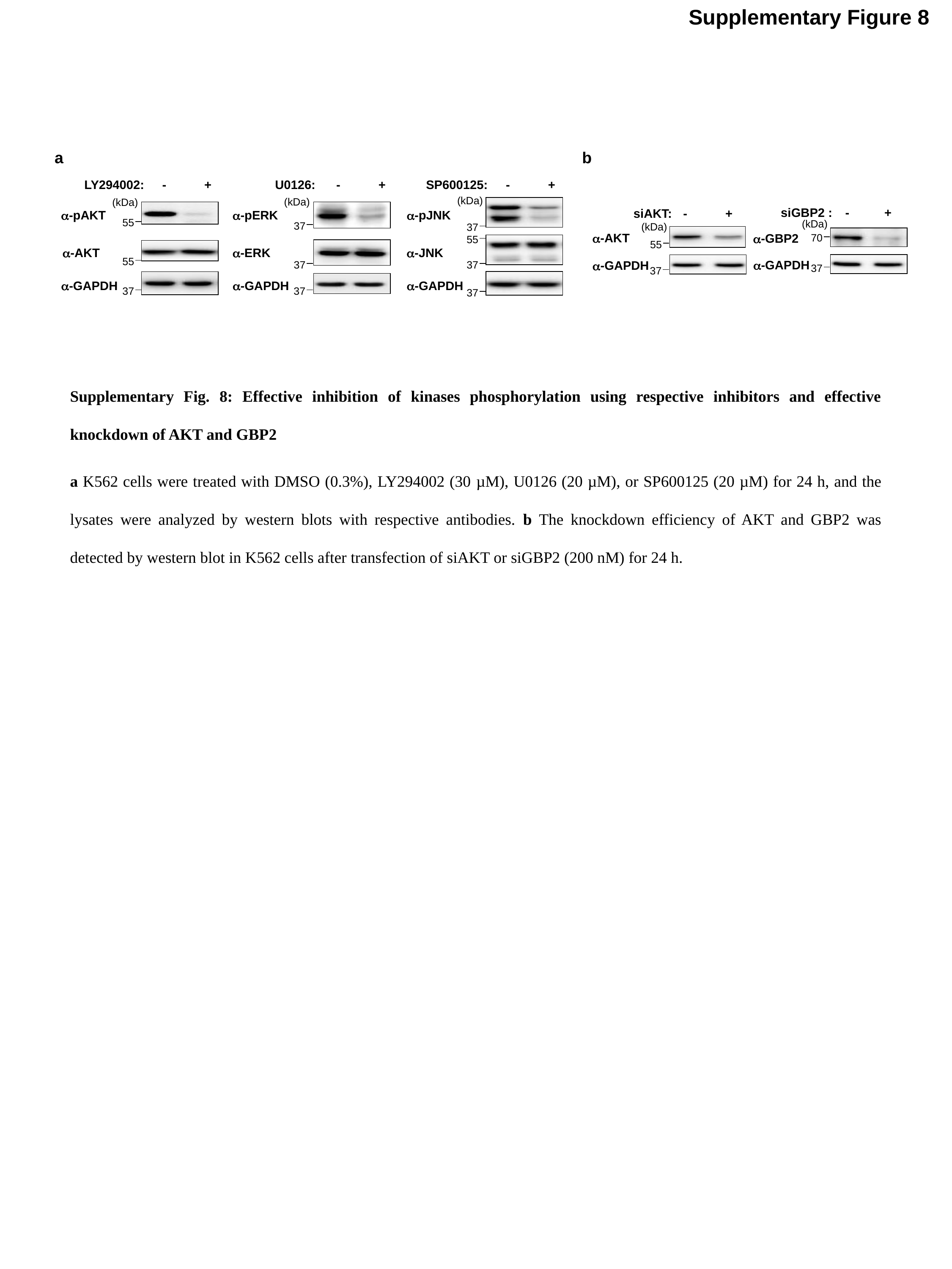

Supplementary Figure 8
a
b
LY294002:
-
+
(kDa)
α-pAKT
55
α-AKT
55
α-GAPDH
37
U0126:
-
+
(kDa)
α-pERK
37
α-ERK
37
α-GAPDH
37
SP600125:
-
+
(kDa)
α-pJNK
37
55
α-JNK
37
α-GAPDH
37
siGBP2 :
-
+
(kDa)
α-GBP2
70
α-GAPDH
37
siAKT:
-
+
(kDa)
α-AKT
55
α-GAPDH
37
Supplementary Fig. 8: Effective inhibition of kinases phosphorylation using respective inhibitors and effective knockdown of AKT and GBP2
a K562 cells were treated with DMSO (0.3%), LY294002 (30 µM), U0126 (20 µM), or SP600125 (20 µM) for 24 h, and the lysates were analyzed by western blots with respective antibodies. b The knockdown efficiency of AKT and GBP2 was detected by western blot in K562 cells after transfection of siAKT or siGBP2 (200 nM) for 24 h.

## Slide 9
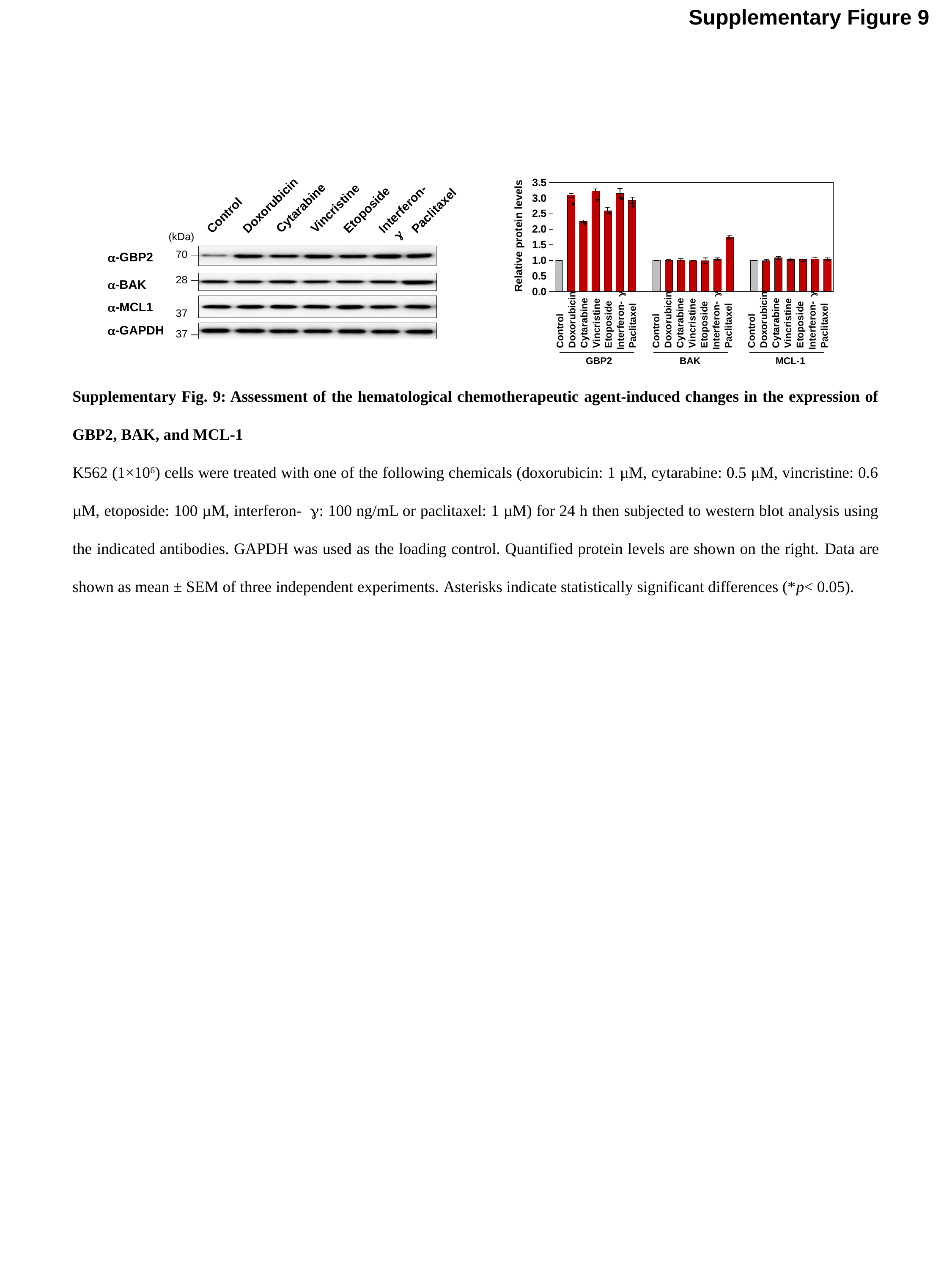

Supplementary Figure 9
### Chart
| Category | |
|---|---|*
*
*
*
*
*
Relative protein levels
*
Doxorubicin
Doxorubicin
Doxorubicin
Interferon- γ
Interferon- γ
Interferon- γ
Cytarabine
Cytarabine
Cytarabine
Vincristine
Vincristine
Vincristine
Etoposide
Etoposide
Etoposide
Paclitaxel
Paclitaxel
Paclitaxel
Control
Control
Control
MCL-1
BAK
GBP2
Control
Doxorubicin
Interferon- γ
Cytarabine
Etoposide
Vincristine
Paclitaxel
(kDa)
70
α-GBP2
28
α-BAK
α-MCL1
37
α-GAPDH
37
Supplementary Fig. 9: Assessment of the hematological chemotherapeutic agent-induced changes in the expression of GBP2, BAK, and MCL-1
K562 (1×10^6^) cells were treated with one of the following chemicals (doxorubicin: 1 µM, cytarabine: 0.5 µM, vincristine: 0.6 µM, etoposide: 100 µM, interferon- γ: 100 ng/mL or paclitaxel: 1 µM) for 24 h then subjected to western blot analysis using the indicated antibodies. GAPDH was used as the loading control. Quantified protein levels are shown on the right. Data are shown as mean ± SEM of three independent experiments. Asterisks indicate statistically significant differences (*p< 0.05).

## Slide 10
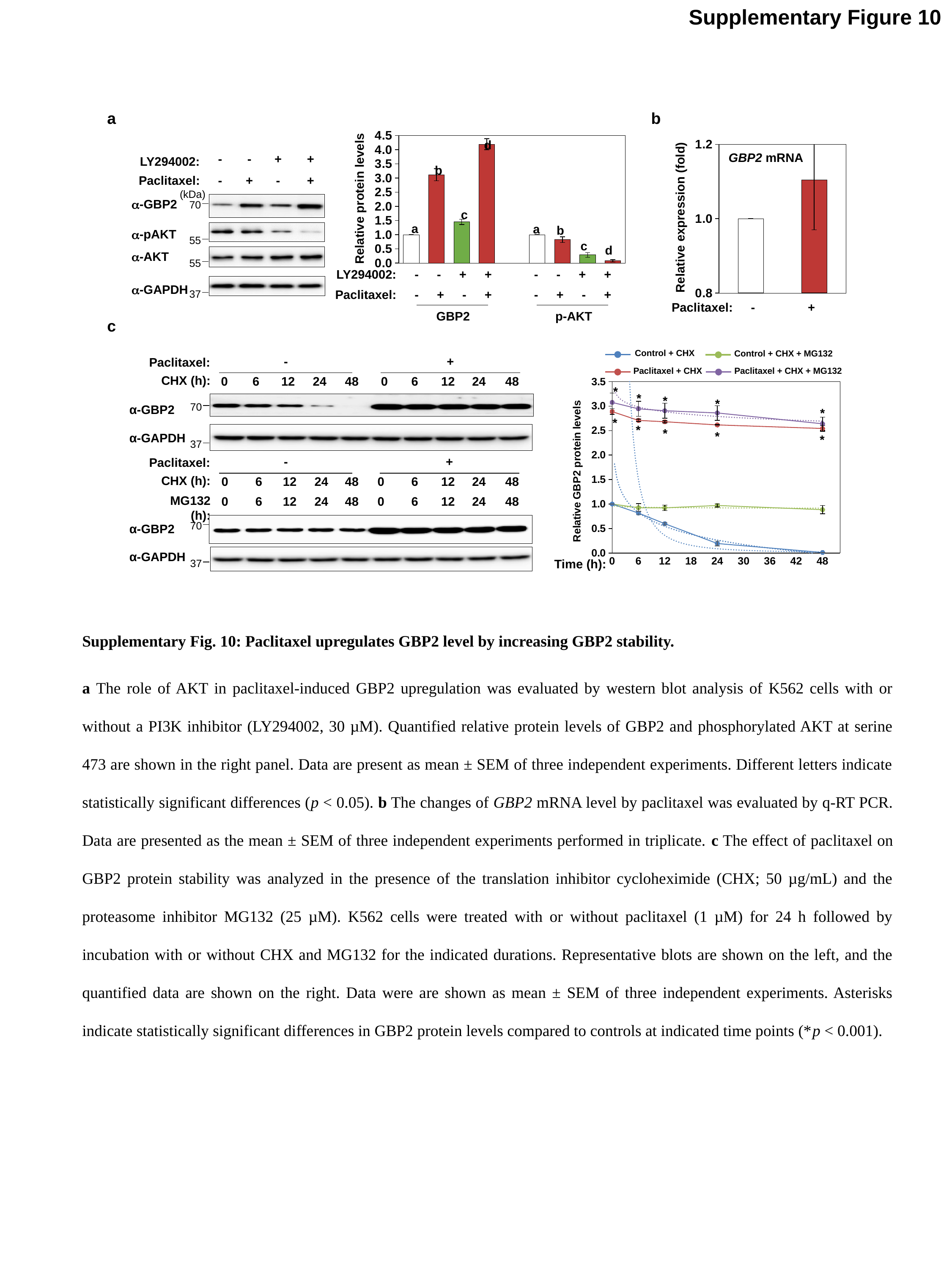

Supplementary Figure 10
a
b
### Chart
| Category | |
|---|---|d
b
Relative protein levels
c
a
a
b
c
d
LY294002:
-
-
+
+
-
-
+
+
Paclitaxel:
-
+
-
+
-
+
-
+
GBP2
p-AKT
### Chart
| Category | |
|---|---|GBP2 mRNA
Relative expression (fold)
+
Paclitaxel:
-
+
-
+
-
LY294002:
+
+
-
Paclitaxel:
-
(kDa)
α-GBP2
70
α-pAKT
55
α-AKT
55
α-GAPDH
37
c
Control + CHX
Control + CHX + MG132
Paclitaxel + CHX
Paclitaxel + CHX + MG132
### Chart
| Category | | | | |
|---|---|---|---|---|*
*
*
*
*
*
*
*
*
*
Time (h):
-
+
Paclitaxel:
CHX (h):
0
6
12
24
48
0
6
12
24
48
70
α-GBP2
α-GAPDH
37
-
+
Paclitaxel:
CHX (h):
0
6
12
24
48
0
6
12
24
48
MG132 (h):
0
6
12
24
48
0
6
12
24
48
70
α-GBP2
α-GAPDH
37
Supplementary Fig. 10: Paclitaxel upregulates GBP2 level by increasing GBP2 stability.
a The role of AKT in paclitaxel-induced GBP2 upregulation was evaluated by western blot analysis of K562 cells with or without a PI3K inhibitor (LY294002, 30 µM). Quantified relative protein levels of GBP2 and phosphorylated AKT at serine 473 are shown in the right panel. Data are present as mean ± SEM of three independent experiments. Different letters indicate statistically significant differences (p < 0.05). b The changes of GBP2 mRNA level by paclitaxel was evaluated by q-RT PCR. Data are presented as the mean ± SEM of three independent experiments performed in triplicate. c The effect of paclitaxel on GBP2 protein stability was analyzed in the presence of the translation inhibitor cycloheximide (CHX; 50 µg/mL) and the proteasome inhibitor MG132 (25 µM). K562 cells were treated with or without paclitaxel (1 µM) for 24 h followed by incubation with or without CHX and MG132 for the indicated durations. Representative blots are shown on the left, and the quantified data are shown on the right. Data were are shown as mean ± SEM of three independent experiments. Asterisks indicate statistically significant differences in GBP2 protein levels compared to controls at indicated time points (*p < 0.001).

## Slide 11
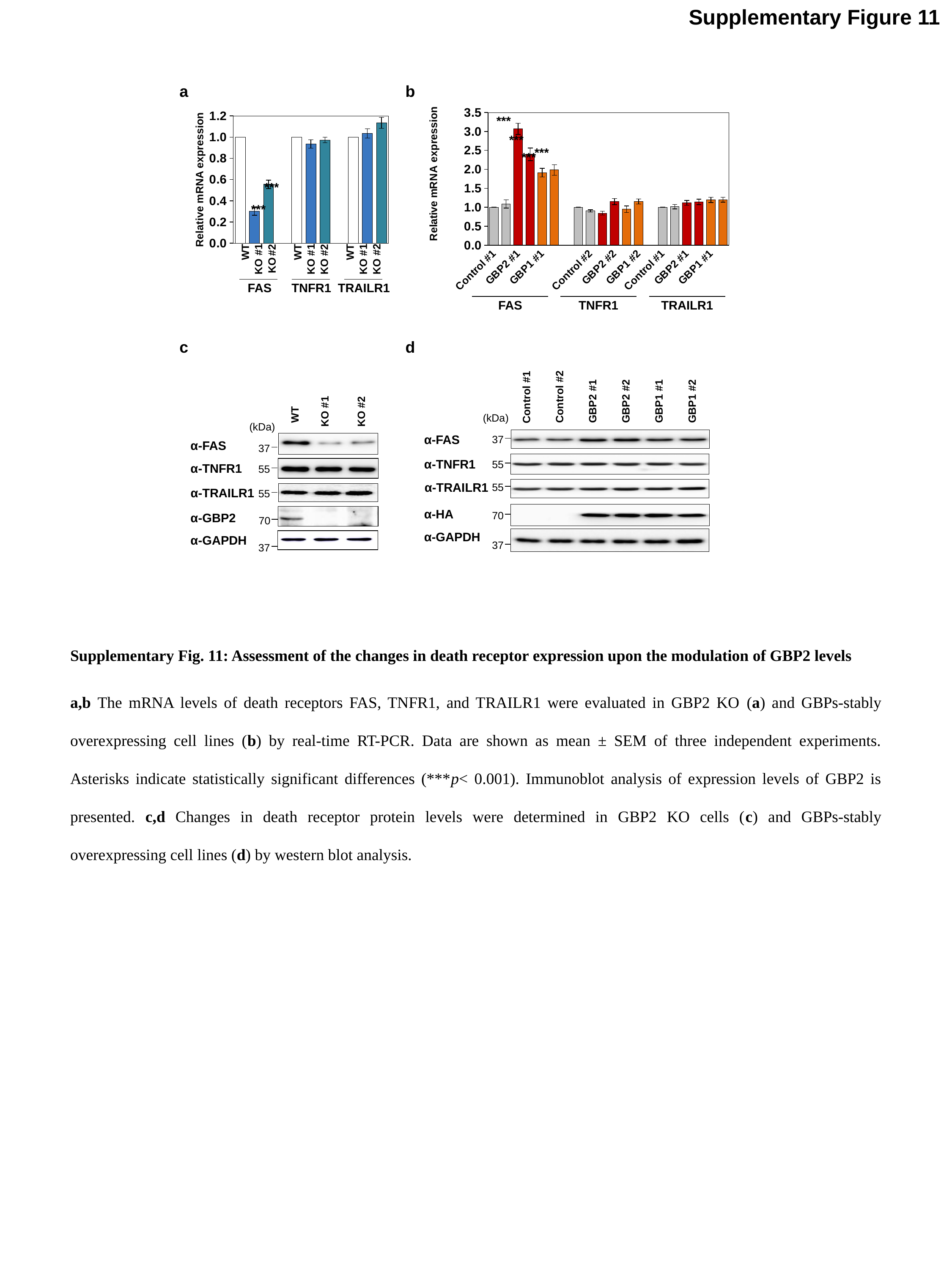

Supplementary Figure 11
a
b
### Chart
| Category | |
|---|---|
| Control #1 | 1.0 |
| Control #2 | 1.0887067511886763 |
| GBP2 #1 | 3.0673788616958344 |
| GBP2 #2 | 2.396883437355577 |
| GBP1 #1 | 1.9144942348762082 |
| GBP1 #2 | 1.9853851331770922 |
| | None |
| Control #1 | 1.0 |
| Control #2 | 0.90311698349167 |
| GBP2 #1 | 0.844692315061533 |
| GBP2 #2 | 1.1496612191958284 |
| GBP1 #1 | 0.9498549710859554 |
| GBP1 #2 | 1.1536163234508634 |
| | None |
| Control #1 | 1.0 |
| Control #2 | 1.0150567952638962 |
| GBP2 #1 | 1.1150182384698288 |
| GBP2 #2 | 1.1403237602896192 |
| GBP1 #1 | 1.1950552003609718 |
| GBP1 #2 | 1.2008438042534193 |
### Chart
| Category | WT |
|---|---|
| WT | 1.0 |
| GBP2-/- #1 | 0.30051964158269945 |
| GBP2-/- #2 | 0.555079146040522 |
| | None |
| WT | 1.0 |
| GBP2-/- #1 | 0.9350399977773538 |
| GBP2-/- #2 | 0.9731573327457725 |
| | None |
| WT | 1.0 |
| GBP2-/- #1 | 1.034449638756076 |
| GBP2-/- #2 | 1.1349400537778427 |***
***
***
***
Relative mRNA expression
***
***
WT
KO #2
KO #1
WT
KO #1
KO #2
WT
KO #1
KO #2
FAS
TNFR1
TRAILR1
FAS
TNFR1
TRAILR1
c
d
Control #1
Control #2
KO #1
KO #2
WT
(kDa)
α-FAS
37
α-TNFR1
55
α-TRAILR1
55
α-GBP2
70
α-GAPDH
37
GBP2 #1
GBP2 #2
GBP1 #1
GBP1 #2
(kDa)
α-FAS
37
α-TNFR1
55
α-TRAILR1
55
α-HA
70
α-GAPDH
37
Supplementary Fig. 11: Assessment of the changes in death receptor expression upon the modulation of GBP2 levels
a,b The mRNA levels of death receptors FAS, TNFR1, and TRAILR1 were evaluated in GBP2 KO (a) and GBPs-stably overexpressing cell lines (b) by real-time RT-PCR. Data are shown as mean ± SEM of three independent experiments. Asterisks indicate statistically significant differences (***p< 0.001). Immunoblot analysis of expression levels of GBP2 is presented. c,d Changes in death receptor protein levels were determined in GBP2 KO cells (c) and GBPs-stably overexpressing cell lines (d) by western blot analysis.
